# Analysis of functional importance of binding sites in the *Drosophila *gap gene network model

**DOI:** 10.1186/1471-2164-16-S13-S7

**Published:** 2015-12-16

**Authors:** Konstantin Kozlov, Vitaly V Gursky, Ivan V Kulakovskiy, Arina Dymova, Maria Samsonova

**Affiliations:** 1 Peter the Great St. Petersburg Polytechnic University, 29 Polytechnicheskaya, 195251 St.Petersburg, Russia; 2 Ioffe Institute, 26 Polytechnicheskaya, 194021 St.Petersburg, Russia; 3 Engelhardt Institute of Molecular Biology, 32 Vavilova, 119991 Moscow, Russia

**Keywords:** transcription, thermodynamics, reaction-diffusion, drosophila

## Abstract

**Background:**

The statistical thermodynamics based approach provides a promising framework for construction of the genotype-phenotype map in many biological systems. Among important aspects of a good model connecting the DNA sequence information with that of a molecular phenotype (gene expression) is the selection of regulatory interactions and relevant transcription factor bindings sites. As the model may predict different levels of the functional importance of specific binding sites in different genomic and regulatory contexts, it is essential to formulate and study such models under different modeling assumptions.

**Results:**

We elaborate a two-layer model for the *Drosophila *gap gene network and include in the model a combined set of transcription factor binding sites and concentration dependent regulatory interaction between gap genes *hunchback *and *Kruppel*. We show that the new variants of the model are more consistent in terms of gene expression predictions for various genetic constructs in comparison to previous work. We quantify the functional importance of binding sites by calculating their impact on gene expression in the model and calculate how these impacts correlate across all sites under different modeling assumptions.

**Conclusions:**

The assumption about the dual interaction between *hb *and *Kr *leads to the most consistent modeling results, but, on the other hand, may obscure existence of indirect interactions between binding sites in regulatory regions of distinct genes. The analysis confirms the previously formulated regulation concept of many weak binding sites working in concert. The model predicts a more or less uniform distribution of functionally important binding sites over the sets of experimentally characterized regulatory modules and other open chromatin domains.

## Background

The construction of a quantitative genotype-phenotype map is one of the most challenging problems in current biology. Mathematical models of gene regulatory networks explicitly connecting the DNA sequence level with that of gene expression or other molecular phenotypic traits represent a flexible framework for studying various aspects of genotype-phenotype mapping [1]. By using this approach, it is possible to assess the importance of various mechanisms involved in translation of genetic information into phenotype and their interplays in different contexts [2,3]. A promising approach for this type of modeling combines methods of the statistical thermodynamics and dynamical systems for model formulation and incorporates the latest experimental results on the sequence and gene expression analysis for its verification [4].

Along this research direction, we previously applied the thermodynamics based model to the *Drosophila *gap gene network, which is a sub-network of the segmentation gene network. The segmentation genes are expressed in early *Drosophila *embryo and determine positions of the body segments [5]. The gap gene sub-network consists of the following genes: *bicoid *(*bcd*) and *caudal *(*cad*) are maternal coordinate genes, *tailless *(*tll*) and *huckebein *(*hkb*) are terminal gap genes, and *hunchback *(*hb*), *Kruppel *(*Kr*), *giant *(*gt*), and *knirps *(*kni*) are trunk gap genes. All these genes encode transcription factors. The model takes as input the potential regulatory regions of trunk gap genes, predicts transcription factor binding sites (TFBSs) and estimates their binding affinities for all transcription factors (TFs) regulating these genes by using positional weight matrices (PWMs), and then calculates as output the dynamics of gene products (both mRNAs and proteins) in the embryo nuclei at the blastoderm stage. The values of free parameters in the model were estimated by fitting the model solution to the *in situ *data on gap gene expression (described in Methods), and the quality of fit was quantitatively satisfactory. We aim to use this model in this study to analyze how functional important TFBSs are distributed in the regulatory regions and how their impacts on gene expression patterns correlate with each other.

A special question of interest is how this analysis depends on specific assumptions about the regulatory interactions between genes. In order to do that, we extend our model in several ways. The first extension concerns new binding sites added to the model. In the first variant of the model, we implemented only those high-affinity TFBSs that fall into the DNase I accessibility regions in the regulatory sequence, as these regions presumably correspond to the open chromatin domains. However, filtering by DNase accessibility might discard some of functional TFBSs, so we added to our model binding sites previously reported as functional (according to the study [6]) but whose coordinates do not intersect with the DNase I accessibility regions.

Another extension concerns new regulatory interactions between *hb *and *Kr *genes. Based on the analysis of fixed mutant expression patterns and *in vitro *data, it has been suggested that Hunchback (Hb) protein acts as a dual regulator of *Kr *: it activates *Kr *at low Hb concentrations and inhibits at high concentrations [7-11]. Based on the previous findings that *Kr *monomer can act as activator, while the homodimer can act as inhibitor [12], a recent study has suggested that a similar dual role (activation at low concentration and repression at high concentration) can be played by Kruppel (Kr) in its regulatory action on *hb*, and a model of this regulatory action is elaborated in that study [13].

The main purpose of this study is to understand the functional organization of the gap gene regulatory regions by developing and applying an advanced model that takes into account the above extensions of our previous model for the gap gene network. We implement both the extended set of TFBSs and the dual regulatory interactions between *hb *and *Kr *in a series of the model variants with increasing complexity. The new variants of the model correctly describe wild-type expression of the four gap genes in the mid-embryo. They also correctly predict gene expression for a larger number of experimentally characterized *cis*-regulatory modules (CRMs) in comparison to the previous model [5]. In the framework of the new model variants, we show that the TFBSs with the strongest impact on gene expressions appear in all domains of the regulatory regions considered in the model. The analysis demonstrates how TFBSs group into clusters of functionally important modules responsible for different spatial parts of the expression patterns, thus forming the *in silico *images of CRMs. We show how TFBSs work in concert by elucidating the correlation between impacts on gene expression from TFBSs of different TFs and from different regulatory regions. Taking all together, the study sheds more light on the binding site organizational level of the gap gene regulatory network.

## Results and discussion

### Selection of binding sites

We predict binding sites using positional weight matrices (PWMs, see Additional file 1) in the region spanning 12 Kbp upstream and 6Kbp downstream of the transcription start site of each of four gap genes *hb*, *Kr*, *kni*, and *gt *and for eight transcription factors Bcd, Cad, Hb, Gt, Kr, Kni, Tll, and Hkb [5]. We apply two constraining factors for binding site selection in order to reduce false-positive predictions. According to the first constraint introduced in the previous work [5], we use only sites belonging to DNase I accessibility regions and not overlapping with the coding sequence. However, this set of sites has only partial overlap with the experimentally verified cis-regulatory modules (CRMs) [6], which drive expression in the trunk region of the embryo at the blastoderm stage, and thus misses a notable fraction of binding sites predictions. In this study, we additionally considered footprint sites and CRMs from RedFly database [14], which is a curated collection of known Drosophila CRMs and TFBSs. The full list of CRMs considered is given in Table 1 and the number of sites for each target gene is shown in Table 2. This new list also includes predicted binding sites which overlap with the TFBSs earlier verified by DNase I footprinting, thus allowing to assess their contribution to the model. The number of sites under consideration is 1419 which is 1.6 times more than 889 sites in the previous work.

**Table 1:** CREs from the RedFly database which drive expression in trunk region of the embryo at the blastoderm stage.

*Hb*	*Kr*	*gt*	*kni*
hb_anterior_activator	Kr_H/I	gt_-10_construct	kni_+1_construct

hb_0.7	Kr_H/J	gt_-1_construct	kni_KSH

hb_HZ340	Kr_CD1	gt_-3_construct	kni_KD

hb_HZ526	Kr_NcS1.7HZ	gt_CE8001	kni_223

hb_HB747	Kr_SN1.7KrZ	gt_gt23	kni_64

hb_distal_minimal	Kr_H/B		kni_223+64

hb_distal_nonminimal	Kr_730		kni_distal

hb_P1 promoter	Kr_H/H		kni_4.4lacZ

hb_HB123	Kr_Kr/A		kni_KBg

hb_HB263	Kr_Kr/D		kni_KC

hb_proximal	Kr_Kr/E		kni_proximal_expanded

hb_pThb1	Kr_Kr/V		kni_KR

hb_HB0.3	Kr_proximal		kni_KT

hb_HB0.8	Kr_dPN5.4KrZ		kni_proximal_minimal

hb_HB4.2	Kr_1BKrZ		kni_KH

hb_matDm0.6-lacZ	Kr_4bcd5KrZ		

hb_matDm0.5-lacZ	Kr_BdelNc0.7HZ		

	Kr_BdelNc0.8HZ		

	Kr_BdelNc1.0HZ		

	Kr_delBNc0.8HZ		

	Kr_delBNc1.0HZ		

	Kr_NsNc1.05HZ		

Total: 17	22	5	15

**Table 2:** Total number of sites used in the model.

	*hb*	*Kr*	*gt*	*kni*
Hb	125	133	111	141

Kr	27	32	44	23

Gt	30	37	36	26

Kni	28	31	33	39

Bcd	38	30	48	36

Cad	35	48	44	52

Tll	28	43	30	32

Hkb	16	7	22	14

Total	327	361	368	363

Columns correspond to target genes, rows to TFs.

### Combined model of gap gene network

In order to explore the functional importance of the selected binding sites in different contexts, we elaborate our previous model of gap gene expression hereinafter referred to as *Model 1 *[5]. It describes the expression of four gap genes in a one-dimensional region on the central midline of the embryo (58 nuclei from 35% to 92% of embryo length, EL), and the time period covering cleavage cycles 13 and 14A that are about 20 min and 50 min long respectively. Cleavage cycle 14A is divided into 8 temporal classes (T1-T8) of 6.5 minutes each. Here, we briefly describe our approach and new incorporated enhancements.

The model derives the gap gene mRNA and protein concentrations from the regulatory sequence. At the first step, we calculate the gene activation probability based on the statistical thermodynamics approach [2]. This approach links the gene activation probability to all possible molecular configurations of the regulatory sequence, representing different combinations of free and bound binding sites. The model incorporates homotypic cooperativity of binding proteins [15] and short range repression [16,17]. At the second step, we use reaction-diffusion equations for the spatio-temporal dynamics of mRNA and protein concentrations of the gap genes *hb*, *Kr*, *gt*, and *kni*, with the time-delay parameter accounting for the temporal separation of transcription and translation processes.

We enhanced the model in three steps with increasing complexity. First, we added the synergy to the model by allowing several DNA bound activators to interact with the basal transcription machinery (BTM) simultaneously ("multiplicative effect" model). The synergistic effects of activators are represented in the previous variant of the model only via the homotypic cooperativity mechanism, while only one bound activator is allowed to directly interact with the BTM in that model ("additive effect" model). It can be shown that the total synergistic effect from all activators will disappear at high activator concentrations under the cooperative binding model (activator binding has already been saturated under this condition, thus cooperative interactions will not be further helpful), but not under the multiplicative model. The maximal number of activators interacting with the BTM is defined as a parameter *N_max_*.

Another improvement in the new variant of the model concerns the way how the solution is calculated. The gene activation probability in *Model 1 *was calculated using the experimental data on protein concentration for TFs Bcd, Cad, Tll, Hkb and also Hb, Kr, Gt, Kni that are included in the model as target genes. The numerical solution of model equations for proteins is used in the new variant of the model as concentration profiles for TFs Hb, Kr, Gt, Kni, as generally expected when simulating differential equations. We use *Model 2 *as the name for the model variant incorporating the synergy and the new simulation method.

Secondly, we added to *Model 2 *the dual action of TF Hb on gene *Kr *by replacing the single parameter for the strength of Hb's action on *Kr *by three parameters: a threshold concentration, an activation strength when Hb concentration is smaller than the threshold concentration, and a repression strength when Hb concentration is larger than this threshold. This variant of the model is called *Model 3*. Finally, we modified *Model 3 *by introducing a similar dual action of Kr on *hb *and call this variant of the model *Model 4*.

Another novelty for *Models 2-4 *in comparison with *Model 1 *concerns the data used for model fitting. The output of *Model 1 *has previously been fitted to the data on gap protein concentrations from the FlyEx database [18,5], while for the new *Models 2-4 *we fit the output both to the protein data and to the RNA data from the SuperFly database [19]. The optimization of parameter values is carried out by the differential evolution (DEEP) method [20] to minimize a combined objective function. This function is a sum of the residual sum of squared differences between the model output and the data, the 'weighted Pattern generating potential' [21], and a penalty term, which limits a growth of regulatory weights (see Methods).

**Figure 1:**
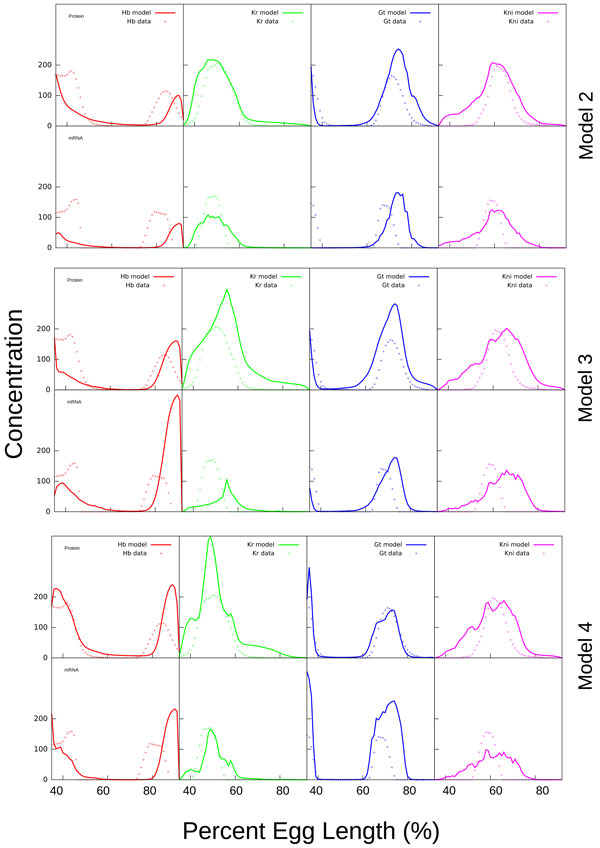
**The output for Models 2, 3 and 4 as compared to protein and mRNA concentration profiles from the FlyEx and SuperFly databases**. Upper and lower rows of panels for each model present results for late (T7) cleavage cycle 14A and for mRNA and protein respectively. Though there are some defects in predicted patterns, all the models correctly reproduce the main features of the system.

We performed about a hundred optimization runs in total for three model variants. It should be noted that several runs are needed due to the stochastic nature of the optimization method in order to find the parameters that provide the minimum value for the combined objective function. We obtained about ten solutions for each model variant with the objective function for proteins close to the value 350000 reported for *Model 1*. The values of combined objective function for these solutions were (75 ± 2) × 10^4^, (75 ± 2) × 10^4 ^and (70 ± 7) × 10^4 ^for *Model 2*, *Model 3 *and *Model 4 *respectively. We select the best model output from optimization runs which both has a small value of the objective function and contains a minimal number of visual defects in the expression patterns. The optimization results for *Models 2-4 *yield solutions of comparable quality as for *Model 1 *(for protein concentrations), and the corresponding gap gene expression patterns qualitatively match the data profiles in the wild type embryo (Figure 1). *Model 4 *provides the best fit among the three new variants of the model. Despite the overall satisfactory correspondence between the solutions and the data, all model variants bear some common inconsistencies in positioning of some domain borders and an expanded Kni domain. On the other hand, the new solutions demonstrate a good balance between the fit qualities for mRNAs and proteins, in contrast to *Model 1 *whose fit quality has only been controlled for proteins. The typical defects in model solution are illustrated in Figure S42.

The regulatory parameters are presented in Tables 3, 4, 5. Most features of the gap gene network topology are in agreement with previous modeling results and data from the literature [22]. Bcd and Cad activate zygotic gap gene expression in a majority of circuits. The only exception is the repression of *gt *by Bcd in *Model 2*. The reciprocal interactions between the trunk gap genes *Kr*, *hb*, *kni*, and *gt *are repressive. Tll represses *Kr*, *gt*, and *kni *and acts as activator of *hb*. Hkb represses *hb*, *Kr*, *gt*, and *kni*. Gt autoactivates itself in all models. Kr and hb autoactivate themselves in *Model 2*, but repress themselves in *Models 3 *and *4*. Kni autoactivates itself in *Models 2 *and *3 *and represses itself in *Model 4*.

**Table 3:** The parameter estimates for Model 2.

Name	Hb	Kr	Gt	Kni	Bcd	Cad	Tll	Hkb
*hb*	86.06	−848.84	−95.87	−748.61	393.44	146.75	5332.19	−6810.68

*Kr*	−830.15	202.90	−1035.26	−582.73	50.25	1102.36	−903.17	−519.44

*gt*	−638.01	−3.19	322.91	−560.23	−2047.91	794.01	−470.24	−286.82

*kni*	−711.85	−614.20	−263.10	250.92	179.91	761.88	−693.47	−378.56

*K*	0.00026	0.019761	0.044755	0.003411	0.049165	0.041029	0.038283	0.008650

*ω*	1.000000	2.114257	4.741914	1.150665	1.000000	5.000000	1.298935	4.366262

*τ*	3.2	3.2	2.2	2.7	Nmax (*q_BTM_*):	2 (3.7 × 10^−6^)	Range:	223

Rows 2-5 present regulatory parameters *T^ab ^*or target genes, while columns correspond to TFs. *K*, *ω *and *τ *are affinity, cooperativity and delay constants respectively. *N_max _* and *q_BTM _* are the number of activators interacting with the BTM and the BTM affinity constant respectively. Range is the range for repressor action in bps.

**Table 4:** The parameter estimates for Model 3.

Name	Hb	Kr	Gt	Kni	Bcd	Cad	Tll	Hkb
*hb*	−380.96	−969.47	−524.70	−362.00	634.59	31.17	17507.75	−31.00

*Kr*	−260.42/4243.26 (10.74)	−280.98	−1862.02	−2151.97	208.23	5019.33	−492.89	−13546.80

*gt*	−2275.97	−438.00	4969.40	−148.88	31.56	603.56	−452.08	−956.16

*kni*	−118.28	−262.29	−2104.13	769.61	13.67	6520.02	−6535.77	−3347.14

*K*	0.052258	0.009202	0.052464	0.035870	0.047500	0.031079	0.044106	0.046209

*ω*	1.000000	2.115275	1.049995	5.152606	1.000000	1.000000	4.992530	2.285720

*τ*	5.8	4.0	1.0	6.8	Nmax (*q_BTM_*):	2 (9.5 × 10^−6^)	Range:	142

Rows 2-5 present regulatory parameters *T^ab ^* for target genes while columns correspond to TFs. The action of *Hb *on *Kr *is characterized by two coefficients for intensities less and greater than the threshold value (given in brackets). *K*, *ω *and *τ *are affinity, cooperativity and delay constants respectively. *N_max _* and *q_BTM _* are the number of activators interacting with the BTM and the BTM affinity constant respectively. Range is the range for repressor action in bps.

**Table 5:** The parameter estimates for model 4.

Name	Hb	Kr	Gt	Kni	Bcd	Cad	Tll	Hkb
*hb*	−425.88	−6524.98/125.80 (13.40)	−517.57	−8608.79	634.59	289.88	1067.94	−265.80

*Kr*	−2148.47/199.57 (52.27)	−280.58	−696.92	−353.16	637.72	465.08	−1374.14	−305.30

*gt*	−1978.04	−3925.54	4839.75	−469.31	14.95	96.84	−565.25	−2996.79

*kni*	−2109.27	−571.96	−459.98	−204.88	11.66	1019.52	−1515.76	−1740.46

*K*	0.000105	0.000103	0.047500	0.047124	0.012524	0.012080	0.049750	0.011963

*ω*	1.000000	1.052592	1.500000	5.233291	1.000000	1.000000	4.713090	1.000075

*τ*	3.5	1.3	7.3	1.0	Nmax (*q_BTM_*):	3 (4.5 × 10^−6^)	Range:	198

Rows 2-5 present regulatory parameters *T^ab ^* for target genes while columns correspond to TFs. The action of *Hb *on *Kr *is characterized by two coefficients for intensities less and greater than the threshold value (given in brackets). *K*, *ω *and *τ *are affinity, cooperativity and delay constants respectively. *N_max _* and *q_BTM _* are the number of activators interacting with the BTM and the BTM affinity constant respectively. The range for the repression is shown in the same row. Range is the range for repressor action in bps.

### Prediction of gap expression in reporter constructs

As the regulatory sequence adopted in the model contains both TFBSs from known CRMs and other potentially strong TFBSs from the accessible chromatin, it is an important test to verify how the CRM's binding sites operate in the background of the rest of the TFBSs. We do it by *in silico *predicting the CRM driven expression in the model and comparing it to the experimental data. We model gap gene expression in reporter constructs by taking as input only those TFBS that overlap with a CRM contained in a reporter [14]. Tables 6, 7, 8 summarize results of sumulations.

**Table 6:** Summary of *hb *and *Kr *expression pattern predictions in reporter constructs by the models (1 and 0 stand for correct and wrong predictions correspondingly).

*Hb*	m2	m3	m4	*Kr*	m2	m3	m4
hb_anterior_activator	1	1	1	Kr_H/I	0	0	0

hb_0.7	1	1	1	Kr_H/J	0	1	1

hb_HZ340	0	0	0	Kr_CD1	1	1	1

hb_HZ526	0	0	0	Kr_NcS1.7HZ	1	1	1

hb_HB747	1	1	1	Kr_SN1.7KrZ	1	1	1

hb_distal_minimal	1	1	1	Kr_H/B	1	1	1

hb_distal_nonminimal	0	0	0	Kr_730	1	1	1

hb_P1 promoter	0	0	1	Kr_H/H	0	0	0

hb_HB123	1	1	1	Kr_Kr/A	1	1	1

hb_HB263	1	1	1	Kr_Kr/D	1	1	1

hb_proximal	1	1	1	Kr_Kr/E	0	0	0

hb_pThb1	1	1	1	Kr_Kr/V	1	1	1

hb_HB0.3	1	1	1	Kr_proximal	1	1	1

hb_HB0.8	1	1	1	Kr_dPN5.4KrZ	1	1	1

hb_HB4.2	0	1	1	Kr_1BKrZ	1	1	1

hb_matDm0.6-lacZ	0	0	0	Kr_4bcd5KrZ	1	1	1

hb_matDm0.5-lacZ	0	0	0	Kr_BdelNc0.7HZ	1	1	1

				Kr_BdelNc0.8HZ	1	0	1

				Kr_BdelNc1.0HZ	1	1	1

				Kr_delBNc0.8HZ	1	1	1

				Kr_delBNc1.0HZ	1	1	1

				Kr_NsNc1.05HZ	1	1	1

Total: 17	10	11	12	22	18	18	19

**Table 7:** Summary of *gt *and *kni *expression pattern predictions in reporter constructs by the models (1 and 0 stand for correct and wrong predictions correspondingly).

*gt*	m2	m3	m4	*kni*	m2	m3	m4
gt_-10_construct	0	0	1	kni_+1 construct	1	1	0

gt_-1_construct	0	0	1	kni_KSH	0	1	1

gt_-3_construct	1	1	1	kni_KD	0	1	1

gt_CE8001	1	1	1	kni_223	1	1	1

gt_gt23	0	0	1	kni_64	1	1	1

				kni_223+64	0	1	0

				kni_4.4lacZ	0	1	1

				kni_KBg	0	1	1

				kni_KC	0	1	1

				kni_KR	0	1	1

				kni_KT	0	1	1

				kni_distal	1	1	1

				kni_proximal_expanded	1	1	1

				kni_proximal_minimal	1	1	1

				kni_KH	0	1	1

Total: 5	2	2	5	15	6	15	13

**Table 8:** Total number of correct predictions of gap gene expression patterns in reporter constructs.

	*hb*	*Kr*	*gt*	*kni*	Total
model2	10	18	2	6	36

model3	11	18	2	15	46

model4	12	19	5	13	49

Columns correspond to target genes, rows to models.

The images of patterns of expression driven by CRMs cloned in reporter constructs and obtained by RNA in situ hybridization with *lacZ *probes are available for the majority of constructs under consideration [14]. From these data we determine what pattern elements of the endogenous gene are driven by the CRM and compare the corresponding parts of data profiles with simulations. We report that prediction is correct if domains of the simulated pattern overlap with those observed experimentally (see Additional files 2, 3, 4, 5).

*Model 4 *has the best prediction power as it successfully predicts gap gene expression patterns in 49 constructs out of 59 in comparison to 36 and 46 correctly predicted by *Model 2 *and *Model 3*, respectively.

Given the fact that the models correctly predict the basic features of gap gene expression in the majority of the constructs we conclude that all the model correctly account for the functional role of most of the included CRM's binding sites. These results also demonstrate the predictive power of the models.

Interestingly, expression patterns driven by some CRMs (e.g. hb_HZ340, hb_HZ526, hb_distal_nonminimal, hb_matDm0.6-lacZ, hb_matDm0.5-lacZ, Kr_HI, Kr_HH, Kr_HE) are not predicted by any model. A possible reason may be that some regulatory mechanisms are still missing in our model, or it is the TFBS prediction quality that limits the model power.

If the expression of the construct is predicted by a model, it is also predicted by subsequent models (models with higher complexity) with 3 exceptions: kni_+1, kni_223+64, Kr_BdelNc0.8. The most deviations in the predictions are for CRMs from the *gt *and *kni *regulatory regions (Table 7): *Models 2 *and *3 *are essentially less accurate than *Model 4 *in *gt *'s CRM simulation, and *Models 3 *and *4 *are more accurate than *Model 2 *in *kni *'s CRM simulation.

### Impact of individual TFBS on model solution

We study the functional importance of TFBSs under different modeling assumptions by calculating the regulatory weight (RW) $w_{r}^{f}$ of a TFBS:

$$w_{r}^{f}=\left(f_{ref}-f_{mut}\right)/f_{ref},$$

where *f *∈ {*RSS,wPGP*} is one of the two measures for the proximity of the solution to the data (see Methods), *f_ref _*is the value of *f *for the model solution for the full set of annotated sites, and *f_mut _*is the same value calculated with the site of interest excluded. Therefore, $w_{r}^{f}$ quantifies the input of the TFBS in the solution. To explore how the regulatory weight depends on the context (either the solution quality assessment or the modeling assumptions), we analyze its values for all model variants, *f *measures and annotated sites.

We find that the RWs estimated with *RSS *and *wPGP *strongly correlate in the case of *Models 2 *and *3 *with Pearson correlation coefficients *r *= 0.85 (*P *= 2.2 × 10^−16^) and *r *= 0.57 (*P *= 2.2 × 10^−16^), respectively (Table 9). The RW does not correlate with the PWM score, a measure of the binding affinity of the site (Table 9). The absence of correlation between the RW measures and PWM scores is not surprising. The later reflects the strength of TF binding *per se *to DNA and proceeds from the premise that neighboring positions in DNA are independent, while the former measure considers the impact of TF removal on phenotype that is mediated by gene interactions in the network. It is worthy of note, that recently the comparative analysis of 12 Drosophila genomes in a phylogenetic framework failed to find evidence that selective constraint across cis-regulatory sequences is correlated with predicted TF binding affinity [23].

**Table 9:** Correlation between different measures of TFBS importance for *Models 2-4 *(from top to bottom, with p-values in parentheses).

	*w^rss^*	*w^wpgp^*
PWM-score	0.08082117 (0.002313)	0.05196682(0.05033)

*w^rss^*	-	0.8484531(< 2.2 × 10^−16^)

PWM-score	0.07118855 (0.007303)	0.09459939(0.0003592)

*w^rss^*	-	0.5713611(< 2.2 × 10^−16^)

PWM-score	0.1553605 (4.03 × 10^−9^)	0.1481884(2.044 × 10^−8^)

*w^rss^*	-	0.2106715(1.1 × 10^−15^)

For *Model 4*, the RWs estimated with *RSS *and *wPGP *have a weaker correlation, *r *= 0.21 (*P *= 1.11 × 10^−15^), but also a bit more essentially correlate with the PWM score, *r *= 0.16 (*P *= 4.03 × 10^−9^) and *r *= 0.15 (*P *= 2.044 × 10^−8^), respectively (Table 9). The loss of correlation between the RWs for the *wPGP *and *RSS *measures indicates that the TFBSs in *Model 4 *encode for those properties of the expression domains which cannot be captured by the standard *RSS *measure. As the *wPGP *measure is shown to be more than the *RSS *one oriented on quantifying the constitutive spatial characteristics of the expression patterns, this result suggests that this variant of the model represents the functional role of TFBSs more accurately. This also appears to be accompanied with the stronger connection between the site impact on expression and its binding affinity, although this connection remains generally weak, in accordance with our previous findings [5].

Interestingly, most of the inter-model correlations are weak or negligible except the one between *w^rss ^*for *Models 3 *and *4*, *r *= 0.28 (*P *= 2.2 × 10^−16^) (see Table 10). The correlation between *w^wpgp ^*for those models is almost twice lower, *r *= 0.16 (*P *= 2.304 × 10^−9^). These results demonstrate that *Models 3 *and *4 *with dual regulatory actions are closer to each other than to *Model 2*.

**Table 10:** Correlations of the TFBS regulatory weights between models.

	model 3 *w^rss^*	model 3 *w^wpgp^*	model 4 *w^rss^*	model 4 *w^wpgp^*
model 2 *w^rss^*	−0.005175102(0.8456)	−0.02373939(0.3715)	0.08631968(0.001135)	0.1424442(7.101 × 10^−8^)

model 2 *w^wpgp^*	−0.04767165(0.07262)	0.001695087(0.9491)	0.003726758(0.8885)	0.1055333(6.804 × 10^−5^)

model 3 *w^rss^*	-	-	0.2870682(< 2.2 × 10^−16^)	0.08221152(0.001939)

model 3 *w^wpgp^*	-	-	0.1176482(8.859 × 10^−6^)	0.1577543(2.304 × 10^−9^)

**Figure 2:**
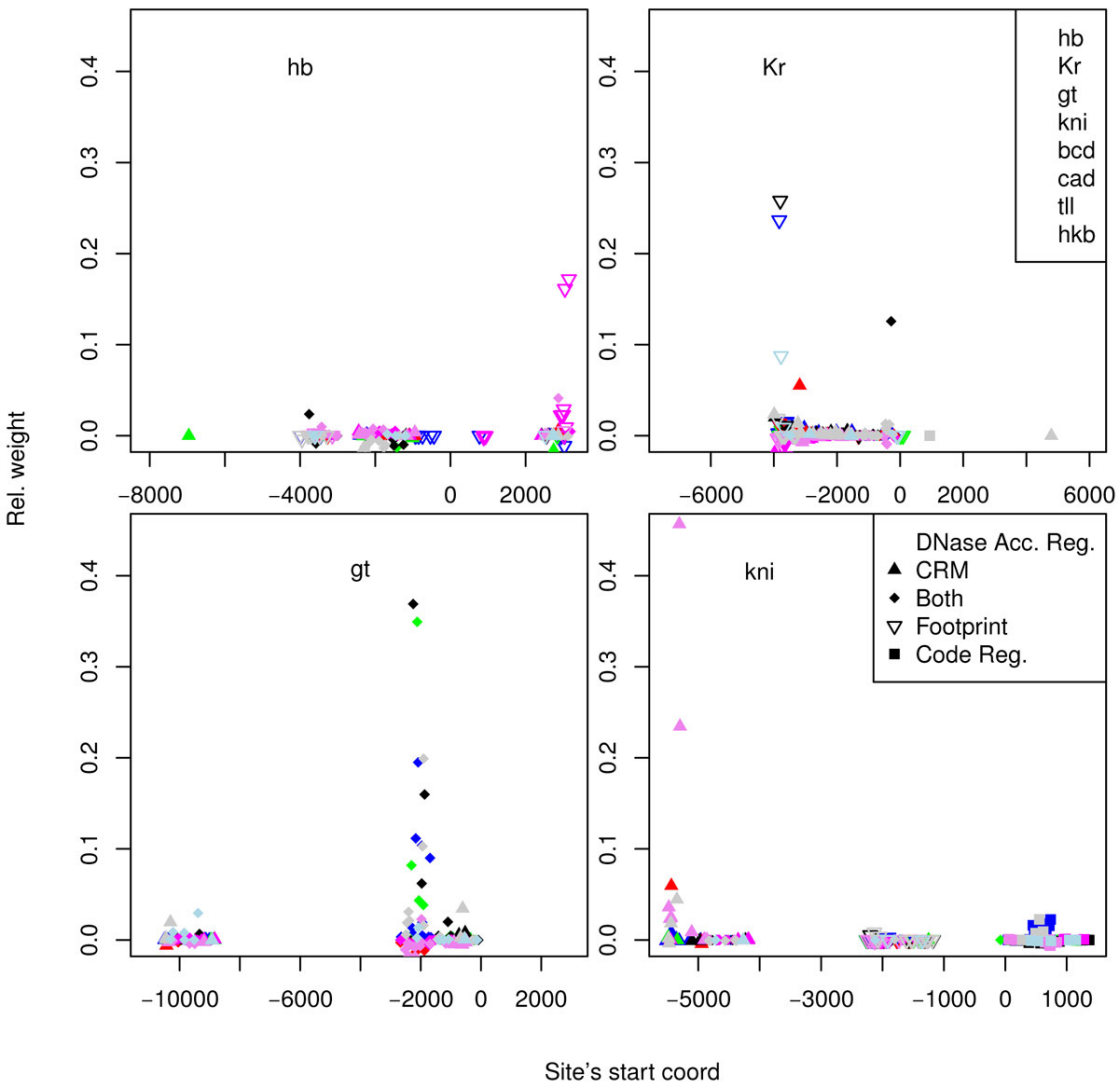
**Plot of the TFBS regulatory weights estimated with the RSS measure and in frame of model 4 relative to site position in a regulatory region**. The binding sites for different TF are shown in different color. The transcription start site is at zero position. Results for *hb *regulatory region are presented relative to TSS of the longest transcript. Sites within CRM are shown as triangles, sites in the DNase I accessible region are marked with circles and rombs present sites in both regions. The empty triangles denote the sites annotated with DNase I footprinting.

In Figure 2, we plot the RWs of TFBSs estimated with the *RSS *measure in *Model 4 *relative to their positions in a regulatory region. Only a small number of sites demonstrate a high impact on the model solution, with the majority of sites having a relatively low individual influence. This finding supports our previous results [5].

**Figure 3:**
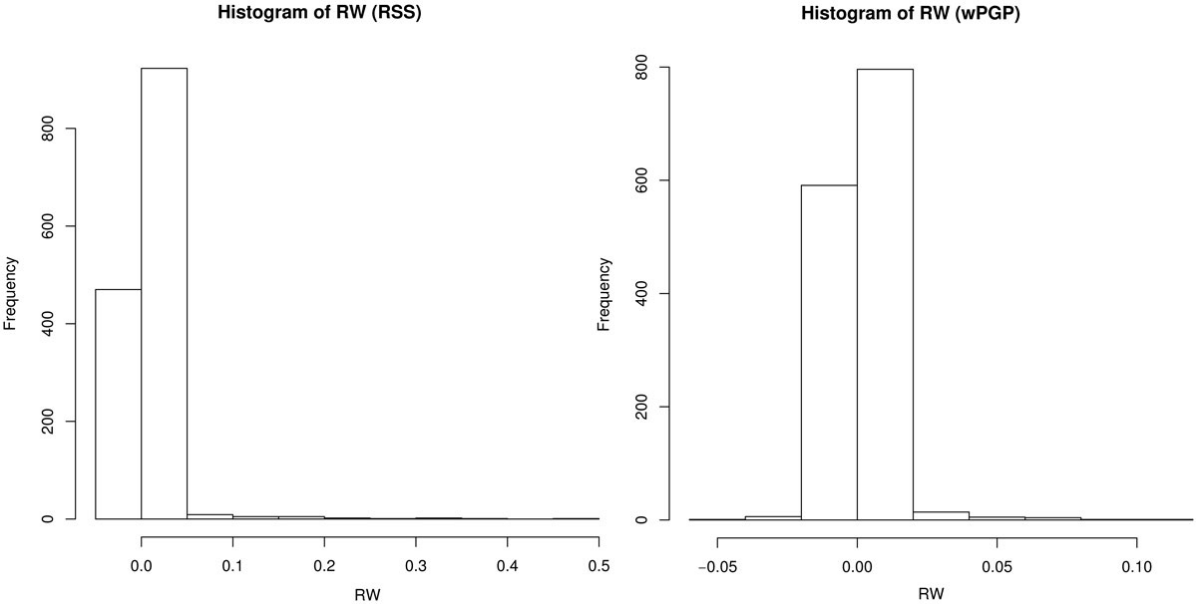
**The histograms of regulatory weights calculted with RSS and wPGP measures**. The thresholds are clearly seen - *w^rss ^*= 0.05 and *w^wpgp ^*= 0.02

Using the histograms of the TFBS RWs (Figure 3), we select the threshold value for *w^rss ^*equal to 0.02 and for *w^wpgp ^*equal to 0.05 and further analyze the sites with *w_r _*exceeding these thresholds. The complete site lists are presented in Tables S9-S19 in Additional file 1. There is a difference between the model variants in the way the strongest sites are distributed between distinct groups of TFBSs, namely belonging to CRMs, DNase I accessibility region, or both (Table 11). In *Model 2*, TFBSs from CRMs play the most important role. All regions contribute approximately similar in *Model 3*. In case of *Model 4*, the majority of important sites belong to both CRMs and the DNase I accessibility region.

**Table 11:** Fraction of high impact sites from different regions.

	CRM	DNase	Both	Total
model2	19	1	14	34

model3	8	9	7	24

model4	13	8	23	44

Columns correspond to regions, rows to models.

Figures 2 and 3 demonstrate that most of the TFBSs are relatively weak and only a few sites are strong in the model with respect to their influence on gene expression. On the other hand, it is not possible to neglect even a small portion of the weak sites without visible reduction in the model output quality (see Figure 7 in [5]). These facts together define the concept of many weak binding sites working in concert, as opposed to the concept of a small number of strong sites controlling everything. It has been shown previously with the help of evolutionary simulations in a similar modeling framework how such enhancer organization may eventually appear during the evolution [24].

### Differential expression of individual TFBS

We further study how the impact of each TFBS on gene expression is distributed in space and time and how these spatio-temporal impact distributions correlate for distinct binding sites. We calculate the impact distribution for a given TFBS by setting the diffusion rate parameter to zero in the model equations and computing the difference in all nuclei and at all times between the solution for the case with all sites included and the solution for the case with the binding site of interest excluded. We do it for all sites and for all new model variants. Setting the diffusion rate equal to zero does not lead to significant perturbations of the expression patterns in the model (Figures S12-S17) and, hence, can be used for the analysis.

**Figure 4:**
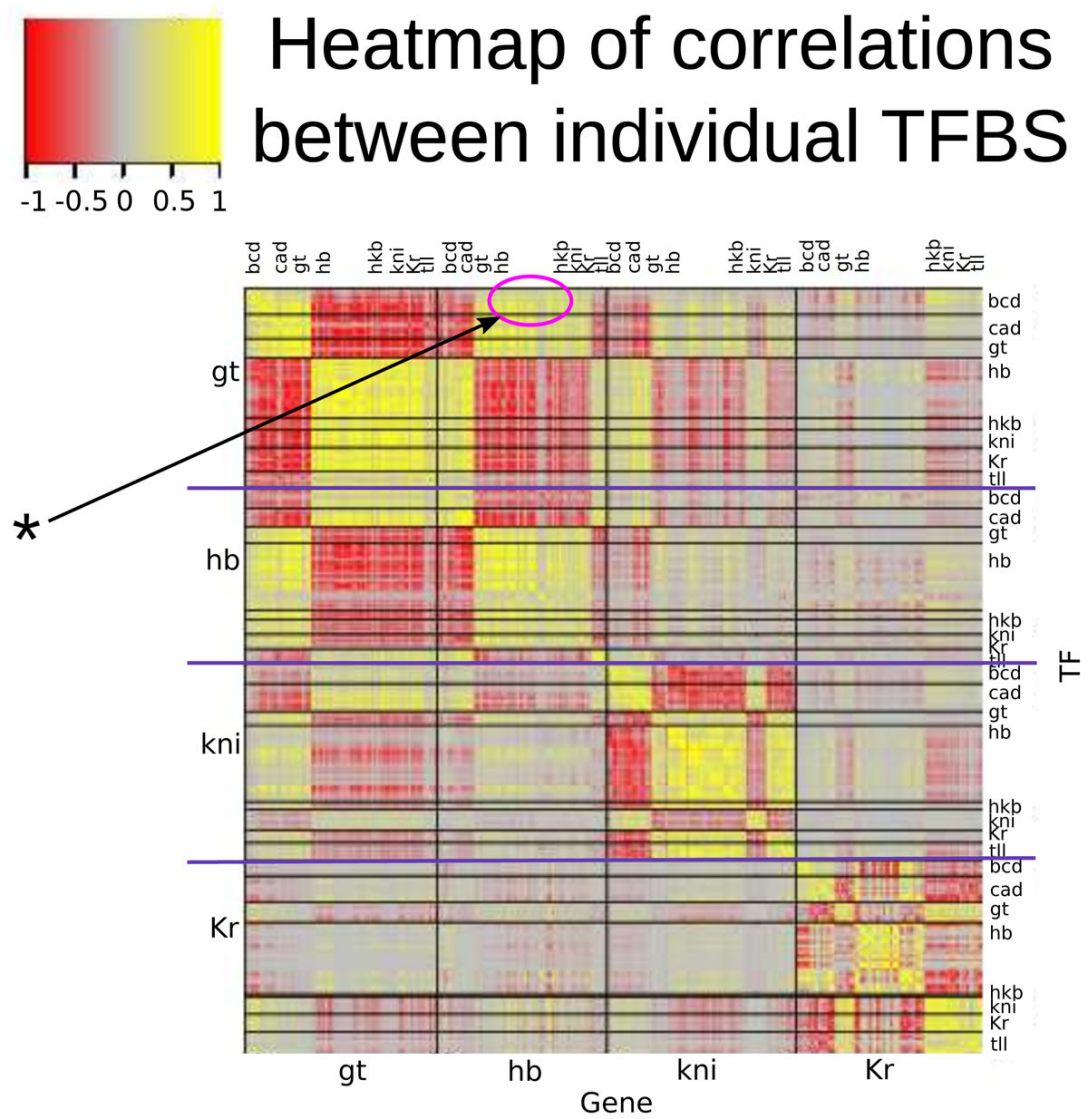
**Correlation matrix between spatio-temporal distributions of TFBS impacts (model 3)**. The diffusion rate parameter was set to zero during calculation. The color in the figure reflects the correlation strength between impact distributions for each pair of TFBSs. The sites are ordered alphabetically - first by target gene (*gt*, *hb*, *kni*, and *Kr*) and then by TF (Bcd, Cad, Gt, Hb, Hkb, Kni, Kr, and Tll) in each group. The clusters of highly correlated sites appear as rectangles of yellow or red color. Arrow with asterisk marks the matrix region which shows high positive correlation between Bcd sites in the gt regulatory region and Hb sites in the *hb *regulatory region.

Figures 4, S27, and S40 show the correlation matrices for the TFBS impact distributions calculated for *Models 3*, *4*, and *2*, respectively. The color in the figure represents the level of correlation between the impact distributions for each pair of TFBSs. If the correlation is positive, the affects of these two TFBSs on gene expression are similar in different nuclei and at different times (either both sites increase expression level or both decrease expression level), i. e. these affects have the same sign. If the correlation is negative, the impacts from the two sites are of different signs across time and space (if one site increases expression level, the other one decreases). The absence of correlation means that for some nuclei and time points the impact from the two sites can be of the same sign, while for others of the opposite signs. The sites are ordered in alphabetical order by target gene (*gt*, *hb*, *kni*, and *Kr) *and, secondly, by TF (Bcd, Cad, Gt, Hb, Hkb, Kni, Kr, and Tll) in each group.

The clusters of highly correlated sites appear in the figure as big square islands of yellow or red color. For *Model 3*, the majority of these clusters are located on the main diagonal and for the regulatory regions of *hb*, *kni*, and *Kr *(Figure 4). This is quite expected result as these TFBS belong to one regulatory region and have either the same or opposite impact on gene expression. In contrast, the Bcd sites in the *gt *regulatory region have high positive correlation with the Hb sites in the *hb *regulatory region (a part of the Figure 4 marked with asterisk). This correlation demonstrates an indirect interaction between the Bcd and Hb binding sites: Bcd activates *gt*, and Gt represses *hb*, so the net effect of the Bcd sites on *hb *is repression, and the same effect is true for the Hb sites on *hb *in *Model 3*. As the clusters of highly correlated sites are mainly located on main diagonal, we conclude that this model variant has low amount of indirect TFBS interactions.

The correlation matrix for *Model 2 *leads to many yellow squares outside of the diagonal, corresponding to highly correlating sites in the regulatory regions of different genes due to indirect interactions (Figure S33). The case of *Model 4 *is somewhere in between with respect to the number of the off-diagonal yellow and red islands (Figure S24). The color intensity of those islands (the correlation strength) is also lower than for *Model 2*. The moderate impact of indirect interactions is intuitive as they are known to be present but are side-effects of the primary regulatory mechanisms. As the number of sites with strong impact is less than 50 for each model, the clusters of sites with high positive or negative correlation can be seen as the illustration of relatively weak impact sites playing in concert. It should also be noted that these indirect effects cannot be captured with *Model 1 *from [5]. This is because, by definition, the indirect connection between a TFBS for a TF *v^a ^*with a TFBS for a TF *v^b ^*takes place only via the influence on another solution component *v^c^*. As mentioned above, the solution in *Model 1 *is calculated by solving equations with the synthesis term that depends only on the data TF profiles at any time, not on the solution, and the data profiles evidently are not influenced by the TFBSs.

**Figure 5:**
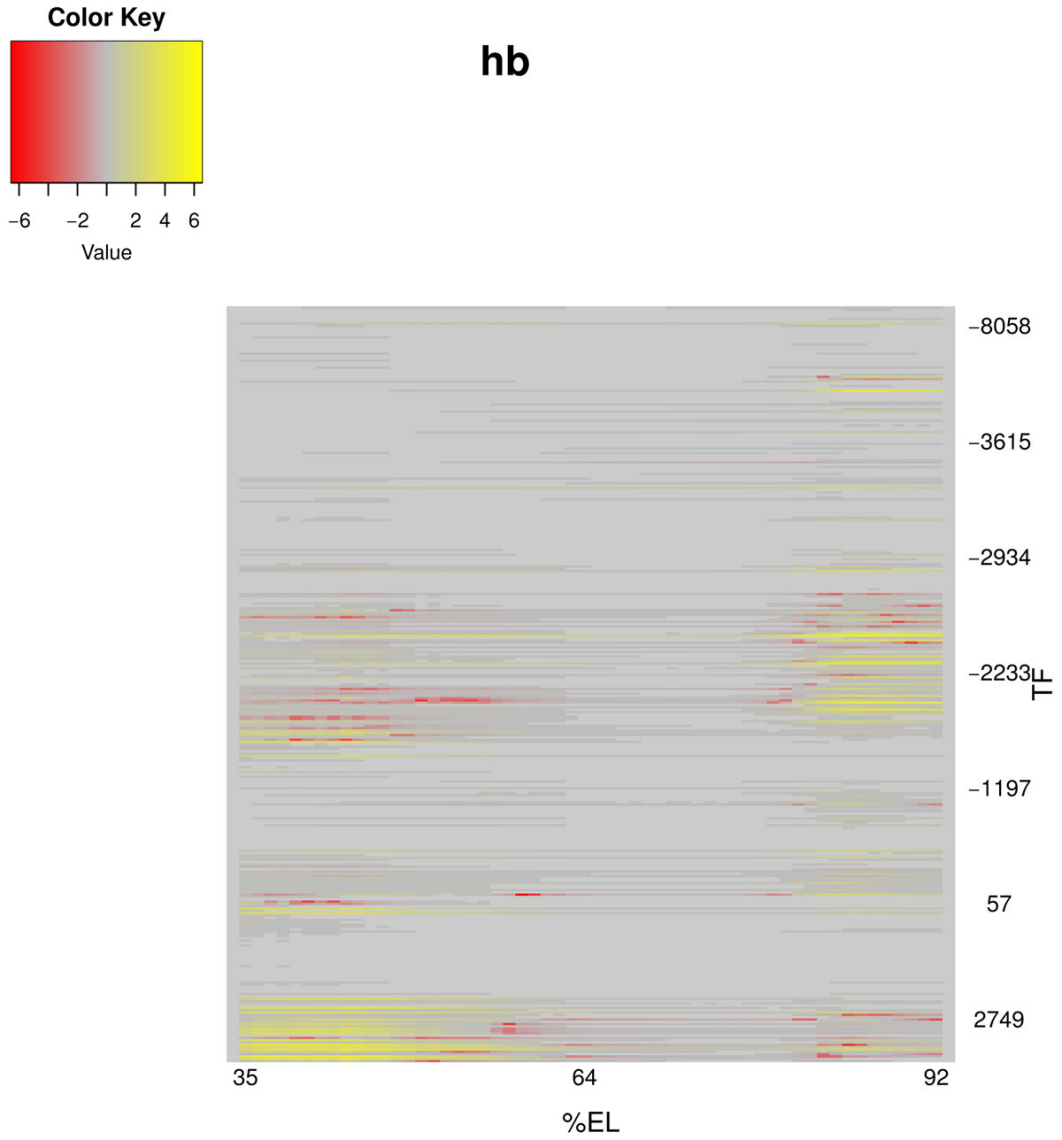
**Spatial distribution of impact on gap gene expression patterns of each TFBS in the hb regulatory region at temporal class 8 (model 4)**. The sites are ordered according to their coordinate. Sites from different parts of the regulatory region modulate expression in different spatial locations. Some visually identifiable clusters of functionally important sites correspond to CRMs, e.g. anteriory expressed CRMs *hb_HB747*, *hb_0.7 *and *hb_0.8 *include sites that cluster at the bottom of this picture.

**Figure 6:**
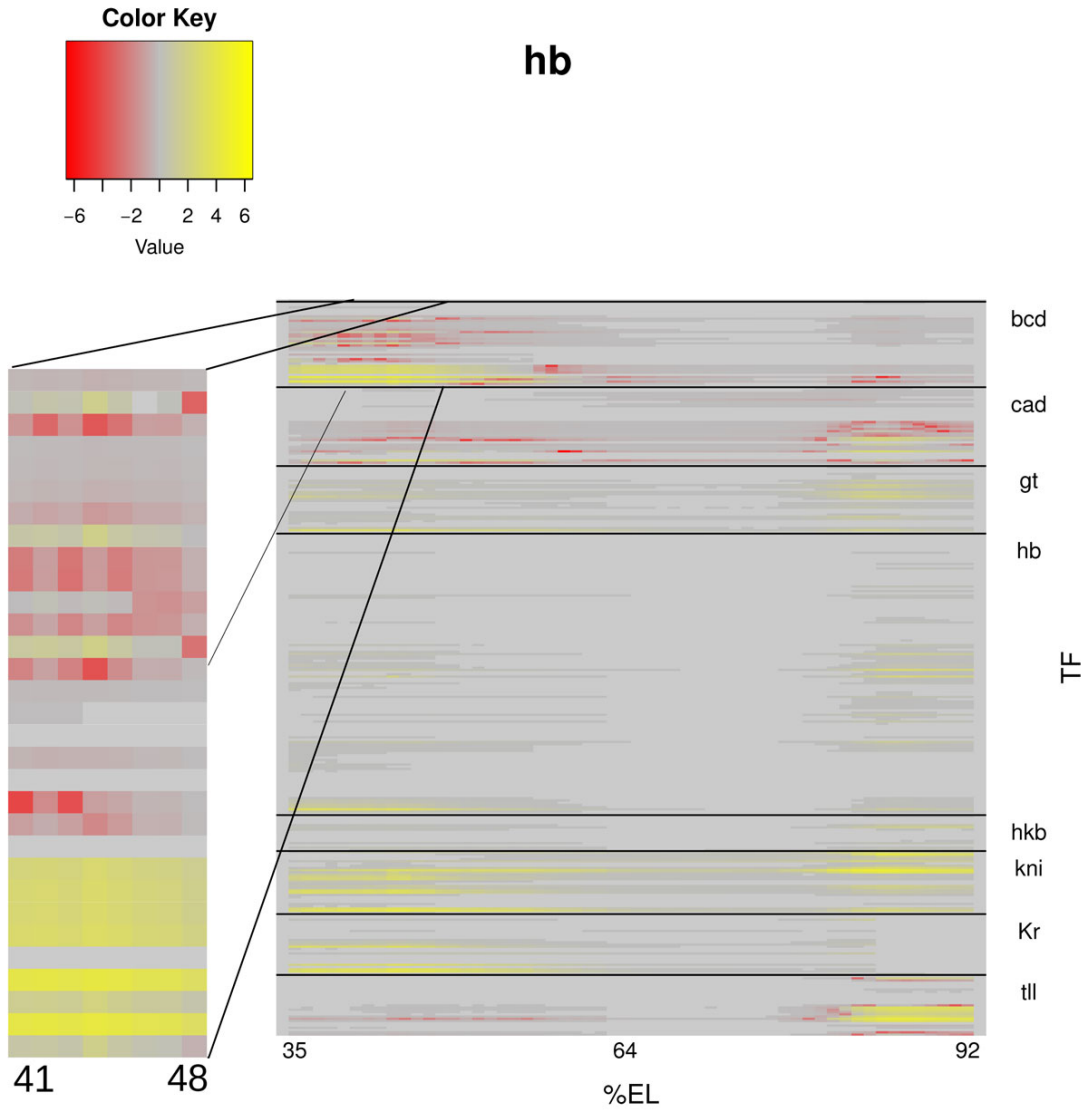
**Spatial distribution of impact on gap gene expression patterns of each TFBS in the hb regulatory region for T8 (model 4)**. The sites are ordered according to the TF and then by coordinate. Different sites of the same TF may have different spatial effects in the model. The insert shows that the strength of site impact can vary between several adjacent nuclei due to regulatory interactions.

Figure 5 presents the spatial distribution of impacts of different TFBSs in *hb *regulatory region on expression of this gene at fixed time point (eighth time class of 14th cleavage cycle, T8 = 67.975) and for *Model 4*. The impact of a site is calculated for each nucleus as log (1 + Δ*u*), where Δ*u *is the normalized difference between the Hb concentrations in the model solution with all sites included and with the site of interest excluded. The negative values of the impact mean that the exclusion of the binding site leads to the increase of Hb concentration in the nucleus (local activating effect from the site), and similarly the positive values mean repressing effect. The sites are ordered along the vertical axis by the local coordinate in the regulatory region relative to the transcription start site (negative values is for upstream, and positive for downstream positions). The horizontal axis corresponds to the spatial domain from 35% to 92% embryo length. Similar plots for other genes (Figures S18, S20, and S22) and model variants (Figures S25, S27, S29, S31, S34, S36, S38, and S40) are presented in Additional file 1. These figures demonstrate how TFBSs from different parts of the regulatory region modulate expression in different spatial locations and may form the CRMs by grouping in local clusters of functional sites. These modules include sites for different TFs (Figure 6; see also Figures S19, S21, and S23 for other genes and Figures S26, S28, S30, S32, S35, S37, S39, and S41 for other model variants).

As opposed to Figure 5, Figure 6 shows the spatial distribution of impacts of different TFs on *hb *expression. Despite the zero diffusion coefficient that prevents the direct transfer of the gene products between the nuclei, the impact of a site may cover several adjacent nuclei with variable magnitude due to regulatory interactions as shown in the inset of Figure 6 by different color intensities for individual nuclei represented by small rectangles.

It can be seen from Figures 5 and 6 that a single TFBS can act both as an activator and a repressor of *hb *depending on the spatial position. For example, some Bcd sites activate *hb *in the posterior domain and repress in the anterior one (Figure 6). This change of the regulation sign may happen only due to interaction with an intermediate TF. As Bcd activates *hb*, the direct impact of its binding sites on *hb *expression can be only activating. However, Bcd also activates all other inhibitors of *hb *(see Table 5), so that its binding sites can indirectly repress *hb *expression in specific positions.

## Conclusions

We presented new modeling results in the framework of the two-layer model for gap gene expression. The previously formulated model [5] had serious drawback, namely the gene activation probability was calculated using the experimental data on TF protein concentration for target genes *hb*, *Kr*, *gt *and *kni*. Here, we introduced several new model variants in which the numerical solution of model equations for proteins is used as concentration profiles for these TFs, as generally expected when simulating differential equations.

Other improvements include the addition of synergy by allowing several DNA bound activators to interact with the BTM simultaneously, dual action of TF Hb on gene *Kr *or dual regulatory interactions between *hb *and *Kr*. All the model variants consider the information about all experimentally characterized CRMs for this developmental system. For all assumptions, the model qualitatively describes the characteristic features of gene expression patterns. However, from the obtained regulatory parameters we may conclude that the assumption about the dual interaction between *hb *and *Kr *leads to the most consistent results.

The defects present in the expression patterns predicted in all variants of the model can have multiple reasons. The thermodynamic modeling approach has its own limitation. For example, it does not take into account the dynamical effects of the enhanceosome assembly [25,26], which might influence the relative probabilities of different molecular configurations of the regulatory regions. The implemented methods for TFBS search may also contain errors, leading to possible mispredictions for binding sites. The imperfect expression pattern for *hb *can be explained by the complex two-promoter structure of this gene, with different enhancers influencing different promoters [27], which currently is not taken into account in our model.

The difference in parameter values between the model variants represents the influence of different modeling assumptions in these variants and cannot be explained by the overfitting or parameter nonidentifiability issues, as we have shown previously that the model is relatively stable in this respect [5]. This difference underlines the disagreement between the models in the predicted correlation patterns for the impacts of TFBSs on gene expression and the functional organization of the regulatory regions. As the model variant with the dual interactions between *hb *and *Kr *(*Model 4*) is more consistent in terms of the topology of the regulatory network and in terms of the predicted CRM's expression, we believe the results obtained for this model variant deserves more attention. However, additional experimental validation is necessary to make a definite decision.

Our results are in agreement with the previously formulated regulation concept of many weak binding sites working in concert [24,5], following the idea of homotypic binding sites clusters [28]. It is hard to define a threshold for the functional importance of binding sites under this concept, and it makes the problem of selecting the complete set of important binding sites more vague. We applied a combined approach for this selection by joining two sets of potential binding sites, high-affinity TFBSs from the open chromatin domain and experimentally characterized TFBSs irrespective of their position in the sequence. Some TFBSs with high functional impact from the *hb*, *Kr*, and *kni *regulatory regions coincide with the strong sites annotated and verified in the DNase I footprint assays. As the majority of functionally important sites in the best model variant belongs to the intersection between the DNase I accessibility region and experimentally characterized CRMs, we conclude that there should be a balance in binding site selection for modeling gene regulation. The existing information about CRMs is essential, but is not enough to fully determine the expression patterns. On the other hand, our results support previous findings about importance of the DNase I accessibility regions usage for modeling [5,29].

Our model allows to estimate the effect of individual TFBS on molecular phenotype, gap gene expression patterns. This effect is mediated by interactions between different bound TFs. An important advantage of new model variants is their ability to account for indirect interactions between individual binding sites. The analysis reveals specific examples of such binding sites in the regulatory regions of the gap genes and elucidates the regulatory mechanism for their interplay. This mechanism provides a potential basis for the evolutionary compensatory effects such as the binding site turnover [30-32]. However, not all variants of the model demonstrate the indirect interactions between the sites. Comparing different modeling assumptions in this context, we conclude that the presence of the dual regulatory action probably obscures existence of such regulatory compensations.

The presented two-layer modeling framework might be applicable to other gene regulatory networks previously described by ODEs alone (e.g [33] (*C. albipunctata*), [34] (*N. vectensis*)) given the data on TFBSs, PWMs and expression patterns are available.

## Methods

### Transcription factor and gene expression data

Concentrations of transcription factors Hb, Kr, Gt, Kni, Bcd, Cad, Tll, and Hkb were taken from FlyEx database (http://urchin.spbcas.ru, [18]), and the mRNA data for those factors were taken from SuperFly database [19]. The resulting data had the form of the gene product concentration profiles in 30-58 nuclei on the anterior-posterior axis of the embryo at nine time points (cleavage cycle 13 and eight time classes in cycle 14A). The gene reporter constructs and their expression images were obtained from REDFly database [14].

### Sequence data

The potential regulatory regions spanning 12 Kbp upstream and 6Kbp downstream of the transcription start site for the gap genes *hb*, *Kr*, *kni*, and *gt *were analyzed, and TFBSs in these regions for transcription factors Bcd, Cad, Hb, Gt, Kr, Kni, Tll, and Hkb were predicted by the method of position weight matrices (PWMs) [35]. The PWMs were described in [36] and can be found at http://www.autosome.ru/iDMMPMM/ (see also the Additional file 1). The PWM thresholds were selected as in [37]. Among all predicted TFBSs, only those were added to the model which satisfy at least one of the following conditions: (1) sites having high PWM score and being located in the DNase I accessibility regions, (2) sites overlapping with the CRMs from the regulatory regions, according to RedFly database [14], and (3) sites overlapping with the TFBSs individually confirmed by DNAse I footprints [14].

### Model equations

The model formulation is presented in our previous paper [5]. Here, we briefly describe the main equations and the modifications introduced in the new study.

The structure of the model equations has two levels. At the first level, the probability of transcriptional activation for each target gene is calculated for each embryo nucleus and at each time moment. At the second level, the dynamics of the mRNA and protein concentrations is prescribed by differential equations which incorporate the activation probability as the synthesis term.

The probability of transcriptional activation is calculated following the thermodynamic approach [2,5]:

(1) $$E_{i}^{a}\left(t\right)=\frac{\sum_{\sigma }W_{i}^{a}\left(\sigma ,t\right)Q^{a}\left(\sigma \right)}{\sum_{\sigma }W_{i}^{a}\left(\sigma ,t\right)Q^{a}\left(\sigma \right)+\sum_{\sigma }W_{i}^{a}\left(\sigma ,t\right)},$$

where *σ *enumerates all possible molecular configurations (sets of free and bound TFBSs) of the regulatory region for gene *a*, $W_{i}^{a}\left(\sigma ,t\right)$ is the statistical weight (relative probability) of configuration *σ *for nucleus-time coordinate (*i, t*), and *Q^a^*(*σ*) includes parameters quantifying the strength of interaction between bound TFs and the basal transcriptional machinery. These weights depend on the concentrations $v_{i}^{b}\left(t\right)$ of all TFs *b *regulating gene *a *in nucleus *i *at time *t*, on the binding affinities of all TFBSs, and other parameters, and corresponding formulas for this dependence are given in [5] and Additional file 1.

The binding affinities of TFBSs are calculated as a part of weights *W *in (1) via the PWM-scores and scaled by a free parameter *K^a^*(*S_max_*), which is the binding affinity constant for the consensus binding site sequence *S_max _*for TF *a*. The possible cooperativity between binding sites is parameterized by the cooperativity constants *ω^a ^*(one constant per each TF *a*) and the distance *d *between the binding sites on which the cooperative interaction is possible. The strength of the influence of bound TF *b *on the target gene *a *is presented in $E_{i}^{a}$ as parameters *T^ab^*, and their negative values correspond to a repressive action, while the positive values to an activating action. The repression is modeled via the short-range mechanism, so that the corresponding parameters *T^ab ^*appear in *W *as weights at molecular configurations with bound repressors. The parameter *d_R _*prescribes the distance in the sequence on which the short-range repression is active in a vicinity of bound repressor. The parameters *T^ab ^*for activators are included in *Q *terms in (1).

The activation probability (1) is translated to differential equations describing the synthesis and transport of mRNAs and proteins. These equations include production, diffusion, and decay terms:

(2) $$du_{i}^{a}/dt=R_{u}^{a}E_{i}^{a}\left(t\right)+D_{u}^{a}\left(n\right)\left[\left(u_{i-1}^{a}-u_{i}^{a}\right)+\left(u_{i+1}^{a}-u_{i}^{a}\right)\right]-\lambda_{u}^{a}u_{i}^{a},$$

(3) $$dv_{i}^{a}/dt=R_{v}^{a}u_{i}^{a}\left(t-\tau_{v}^{a}\right)+D_{v}^{a}\left(n\right)\left[\left(v_{i-1}^{a}-v_{i}^{a}\right)+\left(v_{i+1}^{a}-v_{i}^{a}\right)\right]-\lambda_{v}^{a}v_{i}^{a},$$

where $u_{i}^{a}\left(t\right)$ and $v_{i}^{a}\left(t\right)$ are concentrations of mRNA and protein, respectively, for target gene *a *in nucleus *i *at time *t*. *n *is the cleavage cycle number, $R_{v}^{a}$ and $R_{u}^{a}$ are the maximum synthesis rates, $D_{v}^{a}$ and $D_{u}^{a}$ are the diffusion coefficients, and $\lambda_{v}^{a}$ and $\lambda_{u}^{a}$ are the decay rates for protein and mRNA of gene *a*. The delay parameter *τ *accounts for the average time between events of transcription initiation and corresponding protein synthesis.

The dual transcriptional action of TF *a *on gene *b *is implemented as follows. Instead of a single interaction parameter *T^ab^*, we introduce three: a threshold concentration *V*, an activation strength $T_{+}^{ab}\geq 0$ for the case when *v^a ^*≤ *V *, and a repression strength $T_{-}^{ab}\leq 0$ for the case when *v^a ^*>*V*. This type of transcriptional actions is incorporated in the model for the dual interaction between gap genes *hb *and *Kr*.

### Model fitting

We fitted the model to the mRNA and protein concentration data for gap genes *hb*, *Kr*, *gt*, and *kni*. The values for all free parameters in the model were optimized by the differential evolution entirely parallel (DEEP) method. DEEP is a stochastic global optimization technique described in [20] capable of utilizing several objective functions that combine differential evolution, control of population diversity and the concept of individual age for population member substitution.

The following combined objective function was minimized during the optimization procedure:

$$Error=0.01*RSS+5*10^{4}*wPGP+0.005*Penalty,$$

where the weights were obtained empirically, and the components were defined as follows. The residual sum of squared differences between the model output and data (*RSS*) is calculated and summed up for both mRNA and protein concentrations $u_{i}^{a}\left(t\right)$ and $v_{i}^{a}\left(t\right)$:

$$RSS=\underset{\forall a,i,t:\exists {\text{data}}_{i}^{a}\left(t\right)}{\sum }{\left({\text{model}}_{i}^{a}\left(t\right)-{\text{data}}_{i}^{a}\left(t\right)\right)}^{2}$$

where *a*, *i *and *t *are gene, nucleus, and time point, respectively.

The *weighted Pattern Generation Potential wPGP *was introduced in [21] as a heuristic measure accounting for characteristic features of gap gene expression patterns that it is always less than or equal to 1. We minimize the sum of *wPGP *values calculated separately for mRNA $u_{i}^{a}\left(t\right)$ and protein concentrations $v_{i}^{a}\left(t\right)$ for each gene *a *and time point *t*:

$$wPGP=\underset{\forall a,t:\exists \text{dat}{\text{a}}^{a}\left(t\right)}{\sum }\text{wPG}{\text{P}}^{a}\left(t\right),\;\;\;\;\text{wPG}{\text{P}}^{a}\left(t\right)=\frac{1}{2}+\frac{\text{penalt}{\text{y}}^{a}\left(t\right)-\text{rewar}{\text{d}}^{a}\left(t\right)}{2}$$

where (omitting common variable *t *for time and index *a *for gene)

$$\text{reward}=\frac{\underset{i}{\sum }r_{i}*min\left(r_{i},p_{i}\right)}{\underset{i}{\sum }r_{i}*r_{i}},\;\;\;\;\text{penalty}=\frac{\underset{i}{\sum }\left(r_{max}-r_{i}\right)*|p_{i}-r_{i}|}{\underset{i}{\sum }\left(r_{max}-r_{i}\right)*\underset{i}{\sum }\left(r_{max}-r_{i}\right)}$$

and *p_i _*denotes model output $u_{i}^{a}\left(t\right)$ for mRNA or $v_{i}^{a}\left(t\right)$ for protein while the corresponding data are denoted as *r_i _*with its maximum level *r_max_*. Correctly predicted amount of expression that can be defined for each nuclei as *min*(*p_i_, r_i_*) is weighted by the predicted expression level *r_i _*and 'rewarded' that is subtracted from the *Error*. The incorrect predictions defined as |*p_i _*− *r_i_*| are weighted by the extent of incorrect expression (*r_max _*− *r_i_*) and added to the *Error*, i.e. 'penalized'.

The third component in the combined objective function penalizes the squared values of the regulatory parameters *T^ab^*:

$$Penalty=\underset{\forall a,b}{\sum }{\left(T^{ab}\right)}^{2}$$

This function limits the growth of regulatory parameters, which may have very wide ranges.

## Competing interests

The authors declare that they have no competing interests.

## Authors' contributions

KK and MS formulated the problem, KK, AD, and VG formulated the model, KK, MS, VG, and IVK planned the experiments, KK and AD performed the experiments, IVK and AD calculated PWMs and predicted the TFBSs, KK, AD, MS, VG, and IVK interpreted the results and wrote the paper.

## Supplementary Material

Additional file 1 **Supporting Information**. Positional weight matrices used to predict TFBS, lists of the binding sites with high regulatory impact and additional figures. Click here for file

Additional file 2 **Supporting Information**. Comparison figures. Click here for file

Additional file 3 **Supporting Information**. Comparison figures. Click here for file

Additional file 4 **Supporting Information**. Comparison figures. Click here for file

Additional file 5 **Supporting Information**. Comparison figures. Click here for file

## Declarations

Publication of this article has been funded by the Programme "5-100-2020" by the Russian Ministry of science and education.

This article has been published as part of *BMC Genomics *Volume 16 Supplement 13, 2015: Selected articles from the 7th International Young Scientists School "Systems Biology and Bioinformatics" (SBB'2015): Genomics. The full contents of the supplement are available online at http://www.biomedcentral.com/bmcgenomics/supplements/16/S13.
