# Supplementary material for: Analysis of functional importance of binding sites in the Drosophila gap gene network model
Source: BMC Genomics. 2015 Dec 16;16(Suppl 13):S7. doi: 10.1186/1471-2164-16-S13-S7 (PMC4686791; doi:10.1186/1471-2164-16-S13-S7)
Supplement: Additional file 1 — Supporting Information. Positional weight matrices used to predict TFBS, lists of the binding sites with high regulatory impact and additional figures. [file 1471-2164-16-S13-S7-S1.PDF]

Analysis of Functional Importance of Binding Sites in the Drosophila Gap Gene Network Model. Supporting information

Konstantin Kozlov, Vitaly Gursky, Arina Dymova
Ivan Kulakovskiy and Maria Samsonova

Positional weight matrices

The following PWMs from [1] were used to predict TFBS.

Table S1: PWM for Hb

#	A	C	G	T
1	0.28286611155278	0.592185569462781	-0.136145867625823	-1.76751293532056
2	0.254213296764319	-0.279058161264544	0.435320237208231	-0.718027195298608
3	-3.04079579883957	0.805679778784623	-0.405565047598881	0.250334015567387
4	1.15434963541116	-2.77466891653729	-1.26870528382949	-2.98624111976211
5	1.18718322170129	-2.77466891653729	-2.77466891653729	-2.24344024824139
6	1.18360077649416	-2.65801879597414	-2.77466891653729	-2.1881892028528
7	1.17359485702533	-2.37743934902031	-2.10122450007097	-2.48109811213667
8	1.18891714677379	-2.55935994884054	-2.38229575732011	-2.69963660618354
9	1.09122776392601	-2.16244335534928	-1.62110033494844	-1.31949993562226
10	0.479767371040848	0.413371571223803	-0.553494913191296	-1.13877350496109

Table S2: PWM for Kr

#	A	C	G	T
1	-0.420658339631937	-0.371888097583974	-0.702486856000219	0.665266932827343
2	0.816288527127745	-0.672679826920774	-1.14801634271482	-0.505621250189625
3	1.02514426430819	-0.659463622858145	-2.13702100022947	-1.51520417186872
4	-2.98179845678291	1.32431226437922	-1.87626922286546	-0.620462140165821
5	-1.93735521182007	1.32865041221736	-3.92286969303831	-0.633594128997328
6	-1.76115062471767	1.48853589687916	-3.92286969303831	-3.92286969303831
7	-1.14509405073366	-0.512401154549437	-0.254529726072137	0.762459228355517
8	-1.86579042865168	-2.91908115523225	-1.65663759835415	1.144407069012
9	-3.92286969303831	-0.712178948156372	-3.92286969303831	1.12478690657717
10	-0.936923579599363	0.356401389609112	-0.354030327834424	0.41356829006083
11	0.00312114823627334	-0.123510197304167	0.564176880019257	-0.647751744510587

Table S3: PWM for Gt

#	A	C	G	T
1	0.894743440935813	-2.17380193256571	0.168460518942292	-2.63200259354209
2	0.0132767457861194	-2.15492400269797	-2.89647185910782	0.848580379348455
3	-0.38122444601539	-1.34683914600897	-0.252255198865707	0.706844550786703
4	1.01676970637832	-0.887225390259274	-0.821034544644687	-2.48732253283275
5	-0.714453934227034	1.04746188964819	-1.5648417062544	-0.31660790439989
6	-0.387096908885019	-1.03440516375464	1.21735219487855	-3.18110523181367
7	-1.29646067176372	0.580635090453266	-3.11497935075935	0.615626054743644
8	1.19590645027595	-2.38522993683808	-3.39498210194456	-2.56741818495132
9	1.1657010859841	-1.37216262127687	-2.75061891037223	-3.39498210194456
10	-0.0155498325382931	0.179390117389599	-0.811229269988477	0.247264151388273
11	1.08661227219172	-0.789512730987069	-2.17380193256571	-2.37805752062857
12	0.699718443614972	-0.208588895606494	-0.95528868329782	-0.544997743670269

Table S4: PWM for Kni

#	A	C	G	T
1	0.455726272247978	-0.388552410514004	0.253610849863229	-0.804635777714791
2	0.873682655596035	-1.46330989460881	-0.705451209523316	-0.607039387855736
3	1.17584282015165	-2.22548093796089	-2.28681238211023	-2.52042855490509
4	0.891508245810165	-2.28681238211023	-3.05088857212993	-0.0754907283730982
5	-0.167569112817295	0.433686088340233	-0.264711983511732	-0.0777049616073836
6	-1.12274746895884	-0.77721383498909	-0.181169180578325	0.786479804511023
7	0.794176684153964	-0.922134960951614	0.229047088467897	-3.17158656330019
8	-1.10178205017505	-1.51650811209733	1.38601387202952	-3.48703246125912
9	0.87958610611167	-2.26615431963949	-0.106882407802548	-1.11974612056739
10	-0.360256072608433	-0.54593643631425	0.804746228533211	-0.355643582125967
11	-2.01706986061156	1.43691160020516	-3.37951732164093	-1.5485124623163
12	0.977989859612983	-1.94764360025632	-0.31571613730388	-1.75191459249994
13	0.383982444624124	0.229251479074494	0.149205561377619	-1.50067863561956

Table S5: PWM for Bcd

#	A	C	G	T
1	-0.144456701841385	-1.87077494581996	1.16855734481103	-2.0999731022696
2	-1.30308247972915	-2.25617343877374	1.4136996497966	-2.37517799528534
3	0.917348244882398	0.128638861197661	-2.96357879753955	-2.34412541857431
4	-3.24360785523408	-3.06335363840947	-3.06335363840947	1.21431578153425
5	-2.42596650699202	-3.29887879330889	-3.29887879330889	1.20390064386744
6	1.02820586426514	-2.7190940037466	-1.35260324499878	-0.819932237368865
7	0.1127287152382	-0.902242812585579	0.832931680076778	-1.02203363892937

Table S6: PWM for Cad

#	A	C	G	T
1	-0.244447708833941	-0.0253389851612049	-0.864233992150475	0.508232127797719
2	-1.1762764127624	-1.98444131907814	-1.98444131907814	1.08676754198944
3	-1.08649544118683	-2.58276435352544	-1.64740685796158	1.07865545160057
4	-0.625527203052033	-1.1726928045224	-2.19883783759922	0.967252483469126
5	1.00983507534535	-2.58276435352544	-1.06450225118026	-0.866221178546061
6	-2.74915728201561	-2.58276435352544	-2.58276435352544	1.19406524514381
7	-0.750188864460693	-2.58276435352544	0.885452660984986	0.147546628120431
8	0.617383012458085	-2.6160756958472	0.720585804247287	-2.97727571988906
9	-1.79241413309929	0.622024634223462	0.15081288486018	0.0738539859475068
10	-1.09780598991776	0.713120163981726	-0.741759972965253	0.250863085428389

Table S7: PWM for Tll

#	A	C	G	T
1	0.689610567617782	-0.0452369723117365	-0.70275530984186	-0.886183012468088
2	0.883953461557162	-2.45775857113293	-0.189156750954037	-0.963542181806121
3	1.07938990849982	-0.990361870878966	-1.1667691782418	-3.53391190161659
4	1.0889874099488	-1.09696535299942	-1.16965279225695	-3.53391190161659
5	-1.14300585040928	-2.65377824075658	1.38978467928723	-1.94567641167443
6	-2.0025823039521	-0.150899998985087	-1.92008710225446	0.954814658711214
7	-3.53391190161659	1.38265999439891	-3.53391190161659	-0.725689699525035
8	1.15217258605849	-2.65377824075658	-1.75708800506002	-2.02766810971323
9	1.02179005292275	-3.53391190161659	-0.395421256300573	-1.71859348794298
10	0.546055171807387	0.09224289964789	-0.56441955157107	-0.652847519687621

Table S8: PWM for Hkb

#	A	C	G	T
1	0.212139821249037	-1.45196970854075	0.905491459609423	-1.41993550951193
2	-1.02889935148626	-1.9416251563326	0.658192013508353	0.462111083409141
3	-0.613018052196921	-2.49955348244106	1.3419207356921	-2.99930437854001
4	-2.7933874265196	-2.78806661379028	1.49560608167577	-2.57173903643642
5	-2.79817062558655	-2.50438480827794	1.49817518690456	-2.93131475313798
6	-2.04583529116605	1.45385405050599	-2.19808967776959	-2.23971753352978
7	-2.51597608407632	-2.77528549247748	1.49507371760739	-2.84730474556
8	-3.57861188747833	-3.05142906146399	-0.548080091989963	1.09412471793542
9	-3.05972180654926	-2.33137513542147	0.886752470121085	0.448587514168835
10	0.626498732671432	-1.54956778964025	0.38377563503559	-1.00787259801462

List of binding sites with high regulatory impact in gap gene regulatory regions

Table S9: List of binding sites with high RW in *Kr* regulatory region (model 2)

TF	w^{rss}	w^{wpgp}	Coordinates	construct
cad	0.0342160	0.02806084	21110275	Kr_CD1; Kr_730; Kr_HB; Kr_KrA; Kr_BdelNc0.7HZ; Kr_BdelNc0.8HZ; Kr_BdelNc1.0HZ; Kr_NsNc1.05HZ; Kr_dPN5.4KrZ
cad	0.1170869	0.12646127	21113698	Kr_HI; Kr_SN1.7KrZ; Kr_HB; Kr_HH; Kr_KrA; Kr_KrD; Kr_KrE; Kr_KrV; Kr_dPN5.4KrZ; Kr_proximal
cad	0.1295572	0.13609151	21113723	Kr_HI; Kr_SN1.7KrZ; Kr_HB; Kr_HH; Kr_KrA; Kr_KrD; Kr_KrE; Kr_KrV; Kr_dPN5.4KrZ; Kr_proximal

Table S10: List of binding sites with high RW in *gt* regulatory region (model 2)

TF	w^{rss}	w^{wpgp}	Coordinates	construct
bcd	0.02760357	0.022054522	2327163	
bcd	0.22470970	0.014795591	2325441	gt_CE8001; gt_.3
tll	0.08121365	0.010165633	2325389	gt_CE8001; gt_.3
kni	0.05218994	-0.008191411	2325231	gt_CE8001; gt_.3
cad	0.03606498	0.021916754	2325169	gt_CE8001; gt_.3
bcd	1.05987852	0.412425094	2325129	gt_CE8001; gt_.3
hb	0.07636873	0.031271834	2325070	gt_CE8001; gt_.3
cad	0.04081788	0.023966565	2325003	gt_CE8001; gt_.3
cad	0.36285430	0.102387066	2324960	gt_CE8001; gt_.3
hb	0.06734792	0.028875227	2324946	gt_CE8001; gt_.3
tll	0.06206982	0.009936046	2324920	gt_CE8001; gt_.3
tll	0.22343714	0.048632907	2324891	gt_CE8001; gt_.3
bcd	0.07717895	-0.011456028	2324733	gt_CE8001; gt_.3
bcd	0.58524005	0.395342859	2323666	gt_.1
tll	0.17250814	0.100730835	2323594	gt_.1
kni	0.03176674	0.021089425	2323507	gt_.1

Table S11: List of binding sites with high RW in *kni* regulatory region (model 2)

TF	w^{rss}	w^{wpgp}	Coordinates	construct
Kr	0.456420804	0.0432619959	20693938	
gt	0.054663581	0.0047301012	20693890	
hb	0.100943254	0.0160272865	20693345	
tll	0.125616426	0.0000997336	20688132	kni_.1_construct; kni_proximal_expanded; kni_proximal_minimal
hb	0.132861034	-0.0016693540	20688004	kni_.1_construct; kni_proximal_expanded; kni_proximal_minimal
cad	0.035168188	0.0803099202	20687910	kni_.1_construct; kni_proximal_expanded; kni_proximal_minimal
gt	0.074647602	-0.0009250927	20687868	kni_.1_construct; kni_proximal_expanded; kni_proximal_minimal
cad	-0.004243757	0.0238863026	20687828	kni_.1_construct; kni_proximal_expanded; kni_proximal_minimal
cad	0.054567897	0.0937865025	20687804	kni_.1_construct; kni_proximal_expanded; kni_proximal_minimal
Kr	0.093449104	-0.0033356326	20687765	kni_.1_construct; kni_proximal_expanded; kni_proximal_minimal
gt	0.112438257	0.0016639766	20687759	kni_.1_construct; kni_proximal_expanded; kni_proximal_minimal
cad	0.032872213	0.0770737198	20687736	kni_.1_construct; kni_proximal_expanded; kni_proximal_minimal
cad	0.083916833	0.1145651669	20687720	kni_.1_construct; kni_proximal_expanded; kni_proximal_minimal
Kr	0.104956209	-0.0076706925	20687549	kni_.1_construct; kni_proximal_expanded
Kr	0.054770970	0.0068077384	20687297	kni_.1_construct; kni_proximal_expanded

Table S12: List of binding sites with high RW in *hb* regulatory region (model 3)

TF	w^{rss}	w^{wpgp}	Coordinates	construct
tll	0.05342548	0.02238300	4527482	
bcd	0.05378820	0.03487513	4525003	hb_distal_minimal; hb_distal_nonminimal
bcd	0.06055335	0.03791543	4524960	hb_distal_minimal; hb_distal_nonminimal
Kr	-0.03636123	0.02675322	4524955	hb_distal_minimal; hb_distal_nonminimal
hb	-0.04465588	0.02617683	4520499	hb_anterior_activator; hb_0.7; hb_HB263; hb_HB747; hb_proximal; hb_pThb1; hb_HB0.3; hb_HB0.8; hb_HB4.2
Kr	-0.04943709	0.03058861	4520376	hb_anterior_activator; hb_0.7; hb_HB263; hb_HB747; hb_proximal; hb_pThb1; hb_HB0.3; hb_HB0.8; hb_HB4.2

Table S13: List of binding sites with high RW in *Kr* regulatory region (model 3)

TF	w^{rss}	w^{wpgp}	Coordinates	construct
gt	0.1258432	0.02260763	21110954	Kr_CD1; Kr_HB; Kr_KrA; Kr_BdelNc0.8HZ; Kr_BdelNc1.0HZ; Kr_delBNc0.8HZ; Kr_delBNc1.0HZ; Kr_NsNc1.05HZ; Kr_dPN5.4KrZ
gt	0.2524524	0.03846990	21111160	Kr_CD1; Kr_HB; Kr_KrA; Kr_BdelNc1.0HZ; Kr_delBNc0.8HZ; Kr_delBNc1.0HZ; Kr_NsNc1.05HZ; Kr_dPN5.4KrZ
gt	0.3021682	0.03773047	21113690	Kr_HI; Kr_SN1.7KrZ; Kr_HB; Kr_HH; Kr_KrA; Kr_KrD; Kr_KrE; Kr_KrV; Kr_dPN5.4KrZ; Kr_proximal
kni	0.3308646	0.03270993	21113852	Kr_HI; Kr_SN1.7KrZ; Kr_HB; Kr_HH; Kr_KrA; Kr_KrD; Kr_KrE; Kr_KrV; Kr_dPN5.4KrZ; Kr_proximal

Table S14: List of binding sites with high RW in *gt* regulatory region (model 3)

TF	w^{rss}	w^{wpgp}	Coordinates	construct
cad	0.30361540	0.110206541	2329245	
hb	0.07309241	0.029345171	2329245	
hb	0.09305621	0.035428719	2329212	
hb	0.09061064	0.012673050	2329177	
kni	0.33215173	0.013297178	2329116	
tll	0.20024650	0.042937564	2329051	
kni	0.17319028	0.004779169	2329042	
gt	0.06823387	0.111552348	2329012	
hb	0.12178729	0.016546421	2320014	

Table S15: List of binding sites with high RW in *kni* regulatory region (model 3)

TF	w^{rss}	w^{wpgp}	Coordinates	construct
gt	0.08739398	-0.01848013	20693890	
tll	0.12208900	0.01122684	20693801	
cad	0.02708155	0.03249105	20693761	
cad	0.04653584	0.03904138	20693751	
cad	0.05790861	0.04362843	20693560	

Table S16: List of binding sites with high RW in *hb* regulatory region (model 4)

TF	w^{rss}	w^{wpgp}	Coordinates	construct
bcd	0.1612818	0.04905368	4520486	hb_anterior_activator; hb_0.7; hb_HB263; hb_HB747; hb_proximal; hb_pThb1; hb_HB0.3; hb_HB0.8; hb_HB4.2
bcd	0.1718741	0.05939802	4520381	hb_anterior_activator; hb_0.7; hb_HB263; hb_HB747; hb_proximal; hb_pThb1; hb_HB0.3; hb_HB0.8; hb_HB4.2

Table S17: List of binding sites with high RW in *Kr* regulatory region (model 4)

TF	w^{rss}	w^{wpgp}	Coordinates	construct
bcd	-0.021168563	0.023269666	21107081	
bcd	-0.023853568	0.049035454	21107147	
cad	-0.003234235	0.065908568	21110275	”Kr_CD1”, ”Kr_730”, ”Kr_HB”, ”Kr_KrA”, ”Kr_BdelNc0.7HZ”, ”Kr_BdelNc0.8HZ”, ”Kr_BdelNc1.0HZ”, ”Kr_NsNc1.05HZ”, ”Kr_dPN5.4KrZ”
hb	0.236600098	0.026763975	21110306	”Kr_CD1; Kr_730; Kr_HB; Kr_KrA; Kr_BdelNc0.7HZ; Kr_BdelNc0.8HZ; Kr_BdelNc1.0HZ; Kr_NsNc1.05HZ; Kr_dPN5.4KrZ”
kni	0.258003330	-0.056049891	21110337	”Kr_CD1; Kr_730; Kr_HB; Kr_KrA; Kr_1BKrZ; Kr_BdelNc0.7HZ; Kr_BdelNc0.8HZ; Kr_BdelNc1.0HZ; Kr_delBNc1.0HZ; Kr_NsNc1.05HZ; Kr_dPN5.4KrZ”
hkb	0.087489041	-0.034987760	21110363	”Kr_CD1; Kr_730; Kr_HB; Kr_KrA; Kr_1BKrZ; Kr_BdelNc0.7HZ; Kr_BdelNc0.8HZ; Kr_BdelNc1.0HZ; Kr_delBNc1.0HZ; Kr_NsNc1.05HZ; Kr_dPN5.4KrZ”
bcd	-0.023452823	0.021522888	21110383	”Kr_CD1; Kr_730; Kr_HB; Kr_KrA; Kr_1BKrZ; Kr_BdelNc0.7HZ; Kr_BdelNc0.8HZ; Kr_BdelNc1.0HZ; Kr_delBNc1.0HZ; Kr_NsNc1.05HZ; Kr_dPN5.4KrZ”
gt	0.055138272	-0.008278752	21110954	”Kr_CD1; Kr_HB; Kr_KrA; Kr_BdelNc0.8HZ; Kr_BdelNc1.0HZ; Kr_delBNc0.8HZ; Kr_delBNc1.0HZ; Kr_NsNc1.05HZ; Kr_dPN5.4KrZ”
cad	-0.008227389	0.024214808	21113698	”Kr_HI; Kr_SN1.7KrZ; Kr_HB; Kr_HH; Kr_KrA; Kr_KrD; Kr_KrE; Kr_KrV; Kr_dPN5.4KrZ; Kr_proximal”
cad	-0.009436698	0.030239215	21113723	”Kr_HI; Kr_SN1.7KrZ; Kr_HB; Kr_HH; Kr_KrA; Kr_KrD; Kr_KrE; Kr_KrV; Kr_dPN5.4KrZ; Kr_proximal”
kni	0.125641093	-0.015261517	21113852	”Kr_HI; Kr_SN1.7KrZ; Kr_HB; Kr_HH; Kr_KrA; Kr_KrD; Kr_KrE; Kr_KrV; Kr_dPN5.4KrZ; Kr_proximal”

Table S18: List of binding sites with high RW in *gt* regulatory region (model 4)

TF	w^{rss}	w^{wpgp}	Coordinates	construct
hb	0.05695743	-0.0064635468	2333959	
hkb	0.02961176	0.0268741976	2332373	"gt_gt23" "gt_.10"
kni	0.33855886	0.0054951394	2329116	
tll	0.06559629	0.0686820615	2329051	
kni	0.11630371	-0.0072493801	2329042	
tll	0.03099300	0.0274707523	2325389	"gt_CE8001" "gt_.3"
Kr	0.08200855	-0.0144723925	2325291	"gt_CE8001" "gt_.3"
kni	0.36895250	-0.0173867992	2325231	"gt_CE8001" "gt_.3"
gt	-0.04283896	0.0329944678	2325171	"gt_CE8001" "gt_.3"
hb	0.11159878	-0.0039508338	2325151	"gt_CE8001" "gt_.3"
Kr	0.34930588	-0.0170866535	2325099	"gt_CE8001" "gt_.3"
gt	-0.03745347	0.0395047894	2325072	"gt_CE8001" "gt_.3"
hb	0.19505037	0.0011033915	2325070	"gt_CE8001" "gt_.3"
cad	0.02279353	0.0937821850	2324960	"gt_CE8001" "gt_.3"
kni	0.06208124	-0.0085326888	2324948	"gt_CE8001" "gt_.3"
hb	0.10369813	-0.0016589098	2324946	"gt_CE8001" "gt_.3"
gt	-0.02472660	0.0661945518	2324942	"gt_CE8001" "gt_.3"
tll	0.10297534	0.0134805067	2324920	"gt_CE8001" "gt_.3"
tll	0.19917160	0.0238505769	2324891	"gt_CE8001" "gt_.3"
cad	-0.02459598	0.0209839280	2324866	"gt_CE8001" "gt_.3"
kni	0.15980121	-0.0145463013	2324852	"gt_CE8001" "gt_.3"
hb	0.09003856	0.0004484727	2324675	"gt_CE8001" "gt_.3"
tll	0.03461092	0.0307990958	2323594	"gt_.1"
kni	0.07757639	-0.0074316268	2320114	
hb	0.04109301	0.0225446567	2320014	

Table S19: List of binding sites with high RW in *kni* regulatory region (model 4)

TF	w^{rss}	w^{wpgp}	Coordinates	construct
tll	0.01832055	0.04237350	20693898	
gt	0.05955642	-0.01202706	20693890	
tll	0.04455008	0.05457762	20693801	
cad	0.45670841	0.11733622	20693761	
cad	0.23454492	0.07026353	20693751	
tll	0.02253691	0.02751508	20687900	"kni_.1_construct"<br>"kni_proximal_expanded"<br>"kni_proximal_minimal"

Figures

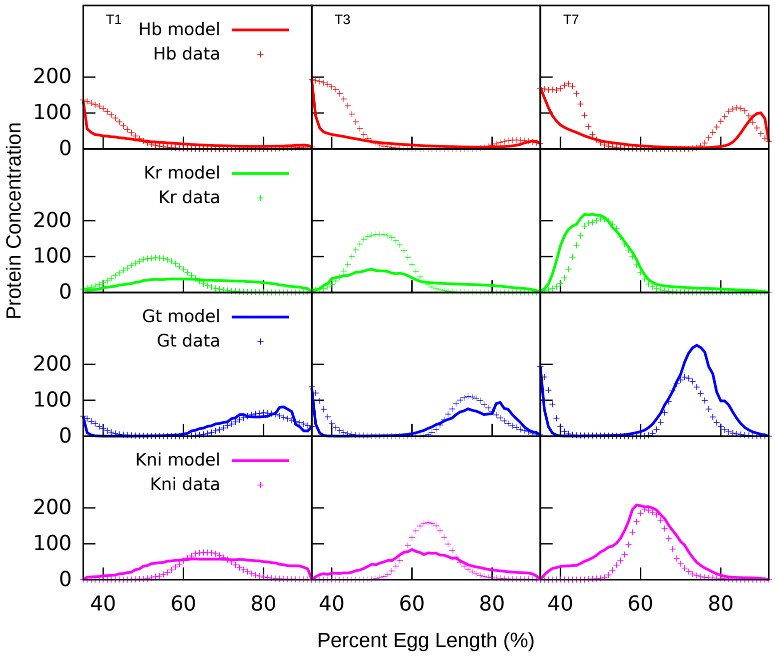

Figure S1: Output for model 2 as compared to protein concentration profiles from the FlyEx. Results are shown for 3 time moments – early (T1), middle (T3) and late (T7) cleavage cycle 14A. Though there are some defects in predicted patterns, the model correctly reproduces the dynamics of the system.

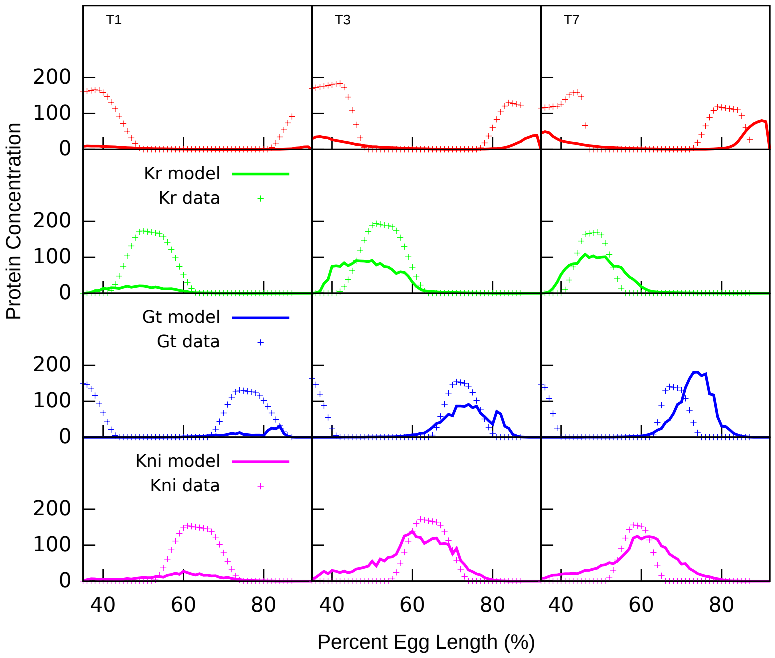

Figure S2: Output for model 2 as compared to mRNA concentration profiles from the SuperFly. Results are shown for 3 time moments – early (T1), middle (T3) and late (T7) cleavage cycle 14A. Though there are some defects in predicted patterns, the model correctly reproduces the dynamics of the system.

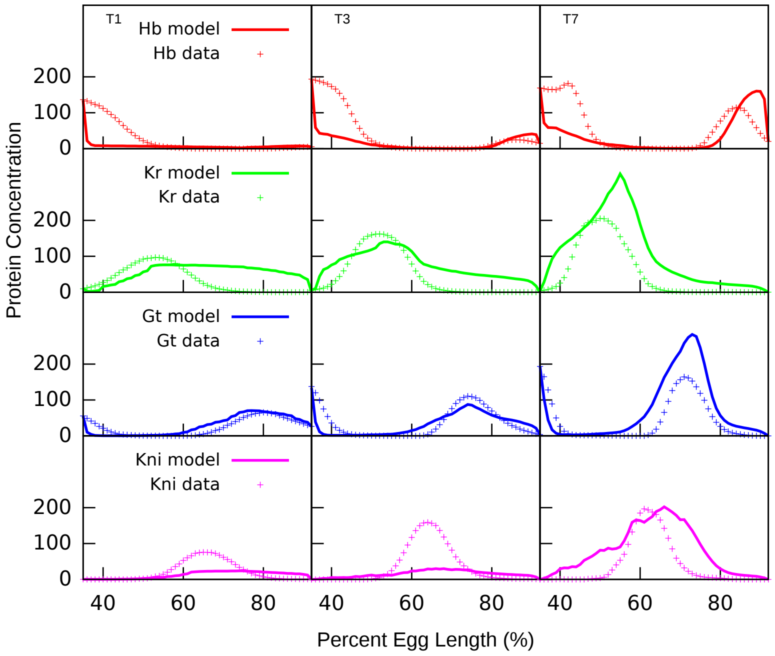

Figure S3: Output for model 3 as compared to protein concentration profiles from the FlyEx. Results are shown for 3 time moments – early (T1), middle (T3) and late (T7) cleavage cycle 14A. Though there are some defects in predicted patterns, the model correctly reproduces the dynamics of the system.

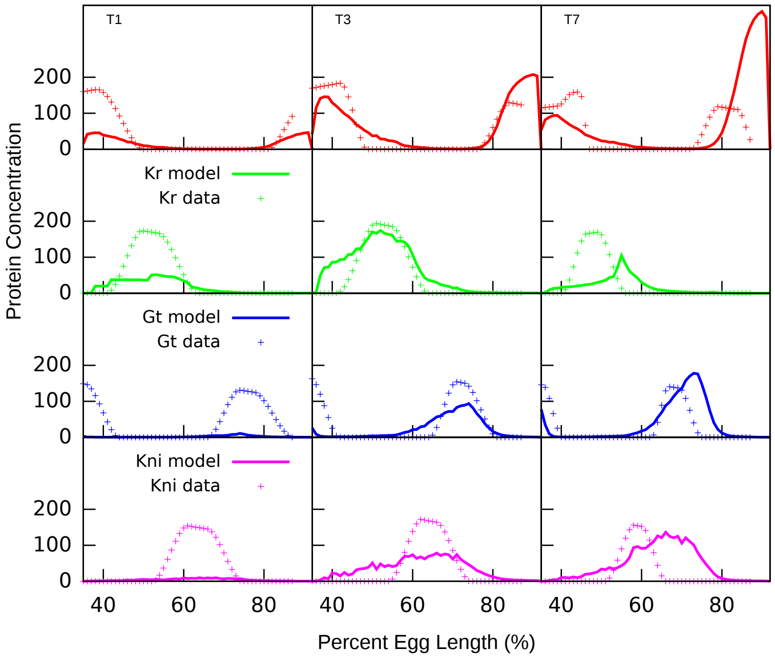

Figure S4: Output for model 3 as compared to mRNA concentration profiles from the SuperFly. Results are shown for 3 time moments – early (T1), middle (T3) and late (T7) cleavage cycle 14A. Though there are some defects in predicted patterns, the model correctly reproduces the dynamics of the system.

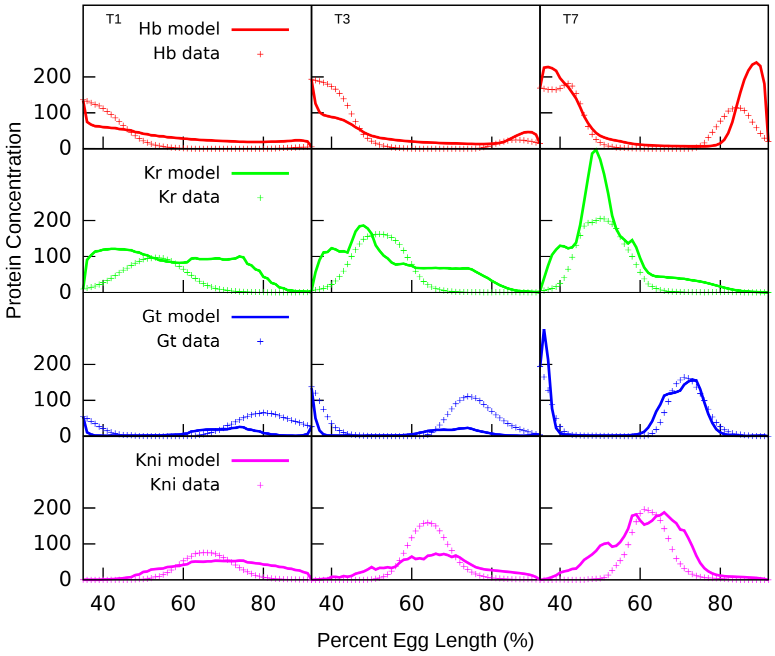

Figure S5: Output for model 4 as compared to protein concentration profiles from the FlyEx. Results are shown for 3 time moments – early (T1), middle (T3) and late (T7) cleavage cycle 14A. Though there are some defects in predicted patterns, the model correctly reproduces the dynamics of the system.

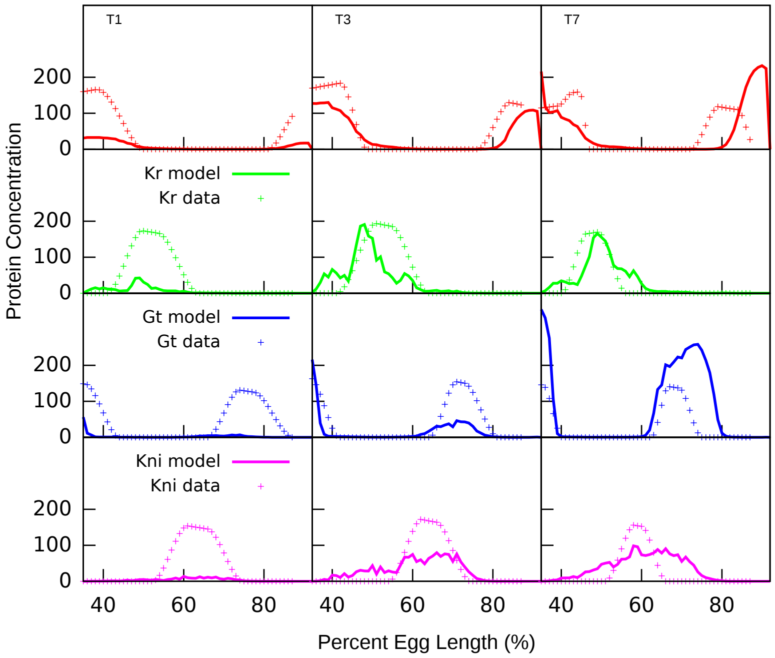

Figure S6: Output for model 4 as compared to mRNA concentration profiles from the SuperFly. Results are shown for 3 time moments – early (T1), middle (T3) and late (T7) cleavage cycle 14A. Though there are some defects in predicted patterns, the model correctly reproduces the dynamics of the system.

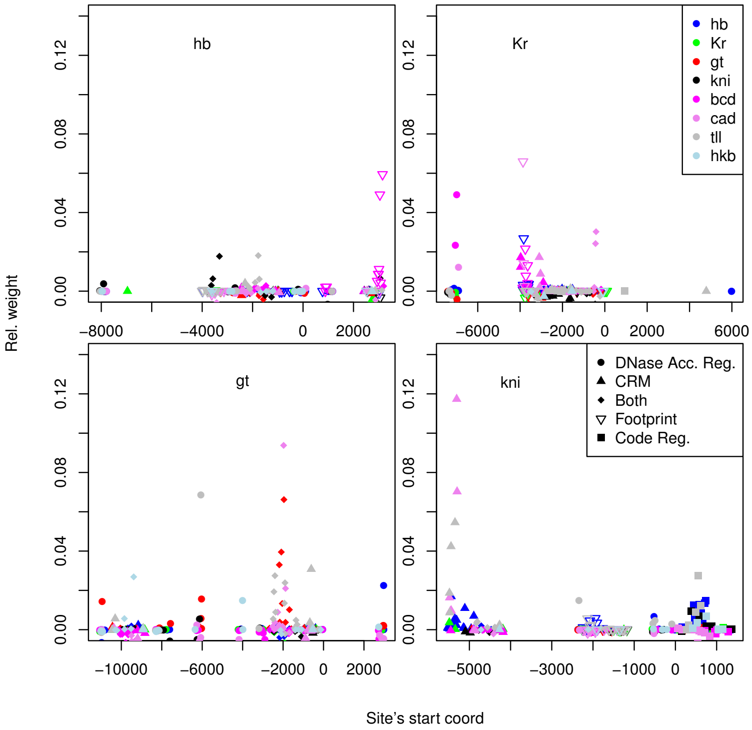

Figure S7: Plot of the TFBS regulatory weights estimated with the wPGP measure and in frame of model 4 relative to site position in a regulatory region. The binding sites for different TF are shown in different color. The transcription start site is at zero position. Results for *hb* regulatory region are presented relative to TSS of the longest transcript. Sites within CRM are shown as triangles, sites in the DNase I accessible region are marked with circles and rombs presents sites in both regions. The empty triangles denote the sites annotated with DNase I footprinting.

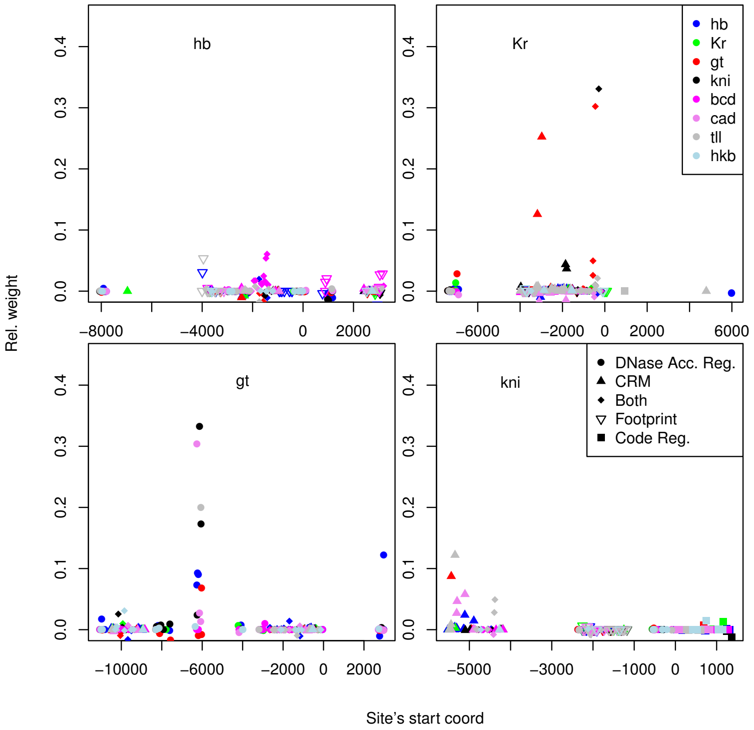

Figure S8: Plot of the TFBS regulatory weights estimated with the RSS measure and in frame of model 3 relative to site position in a regulatory region. The binding sites for different TF are shown in different color. The transcription start site is at zero position. Results for *hb* regulatory region are presented relative to TSS of the longest transcript. Sites within CRM are shown as triangles, sites in the DNase I accessible region are marked with circles and rombs presents sites in both regions. The empty triangles denote the sites annotated with DNase I footprinting.

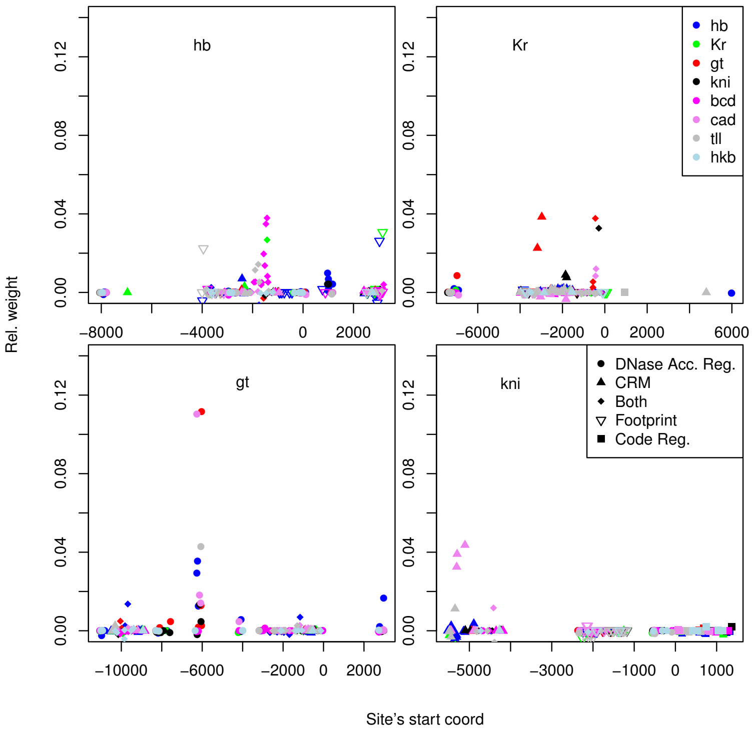

Figure S9: Plot of the TFBS regulatory weights estimated with the wPGP measure and in frame of model 3 relative to site position in a regulatory region. The binding sites for different TF are shown in different color. The transcription start site is at zero position. Results for *hb* regulatory region are presented relative to TSS of the longest transcript. Sites within CRM are shown as triangles, sites in the DNase I accessible region are marked with circles and rombs presents sites in both regions. The empty triangles denote the sites annotated with DNase I footprinting.

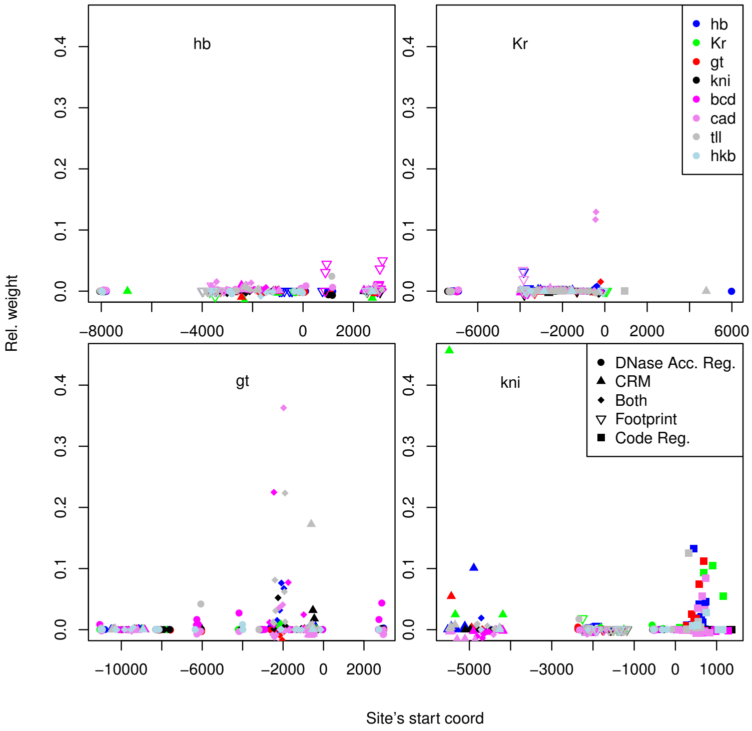

Figure S10: Plot of the TFBS regulatory weights estimated with the RSS measure and in frame of model 2 relative to site position in a regulatory region. The binding sites for different TF are shown in different color. The transcription start site is at zero position. Results for *hb* regulatory region are presented relative to TSS of the longest transcript. Sites within CRM are shown as triangles, sites in the DNase I accessible region are marked with circles and rombs presents sites in both regions. The empty triangles denote the sites annotated with DNase I footprinting.

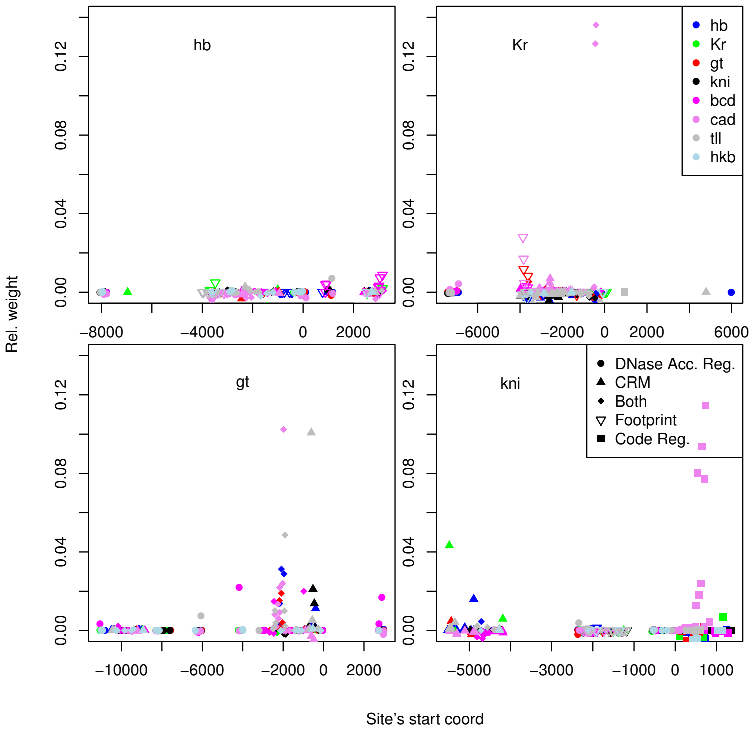

Figure S11: Plot of the TFBS regulatory weights estimated with the wPGP measure and in frame of model 2 relative to site position in a regulatory region. The binding sites for different TF are shown in different color. The transcription start site is at zero position. Results for *hb* regulatory region are presented relative to TSS of the longest transcript. Sites within CRM are shown as triangles, sites in the DNase I accessible region are marked with circles and rombs presents sites in both regions. The empty triangles denote the sites annotated with DNase I footprinting.

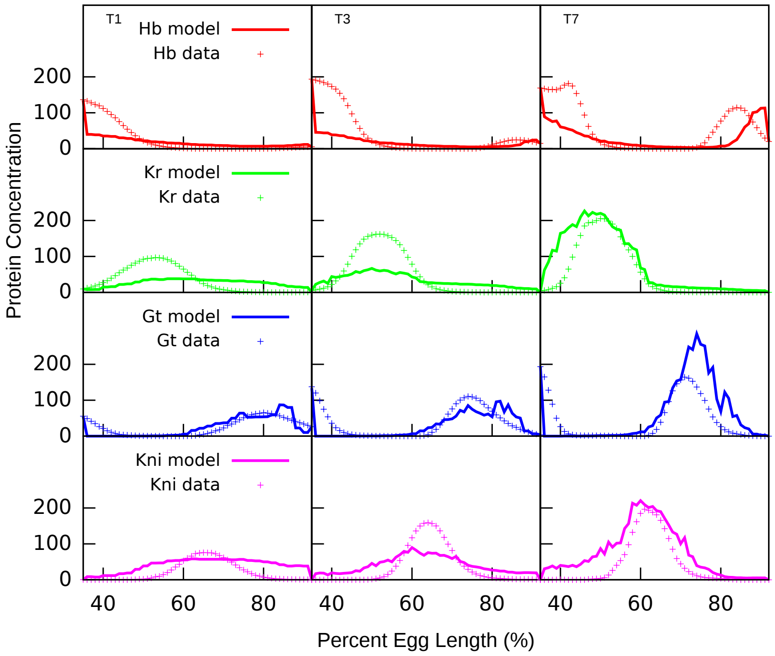

Figure S12: Output for model 2 w/o D as compared to protein concentration profiles from the FlyEx. Results are shown for 3 time moments – early (T1), middle (T3) and late (T7) cleavage cycle 14A.

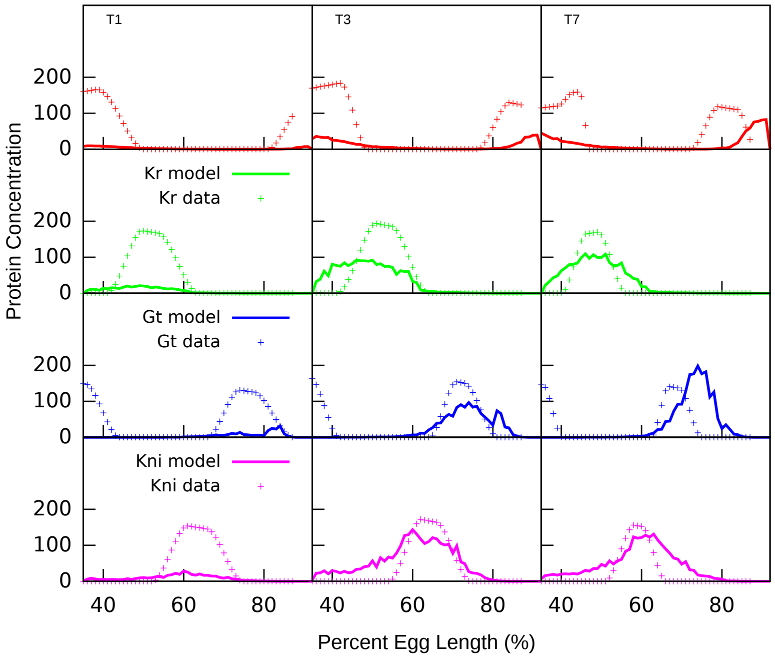

Figure S13: Output for model 2 w/o D as compared to mRNA concentration profiles from the SuperFly. Results are shown for 3 time moments – early (T1), middle (T3) and late (T7) cleavage cycle 14A.

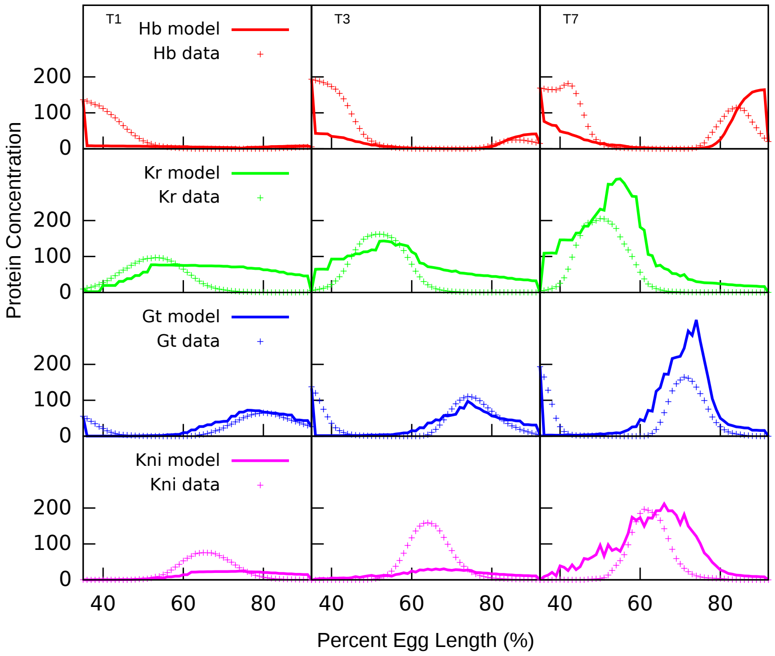

Figure S14: Output for model 3 w/o D as compared to protein concentration profiles from the FlyEx. Results are shown for 3 time moments – early (T1), middle (T3) and late (T7) cleavage cycle 14A.

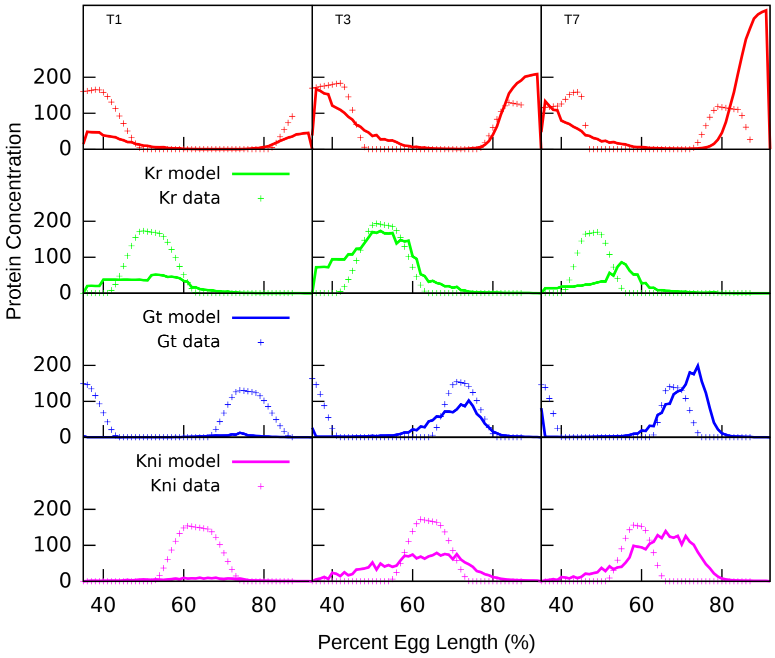

Figure S15: Output for model 3 w/o D as compared mRNA concentration profiles from the SuperFly. Results are shown for 3 time moments – early (T1), middle (T3) and late (T7) cleavage cycle 14A.

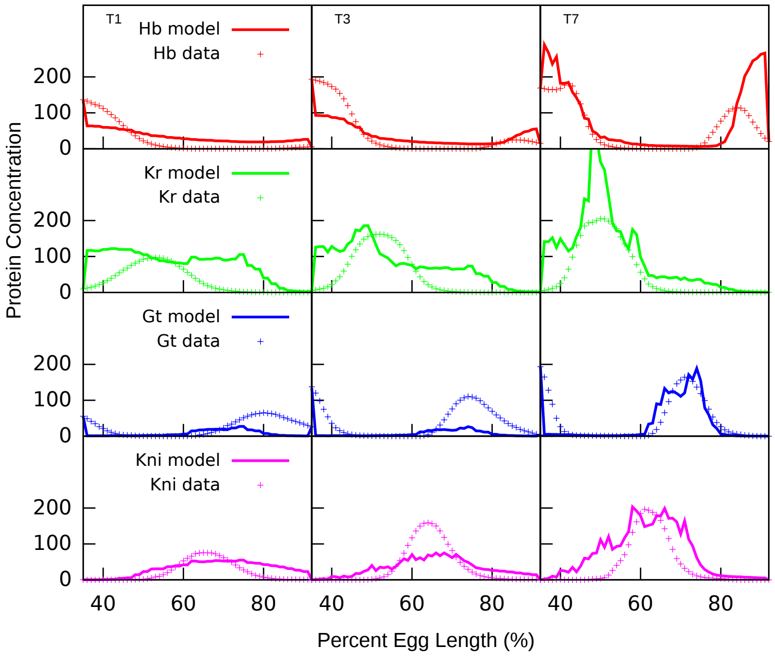

Figure S16: Output for model 4 w/o D as compared to protein concentration profiles from the FlyEx. Results are shown for 3 time moments – early (T1), middle (T3) and late (T7) cleavage cycle 14A.

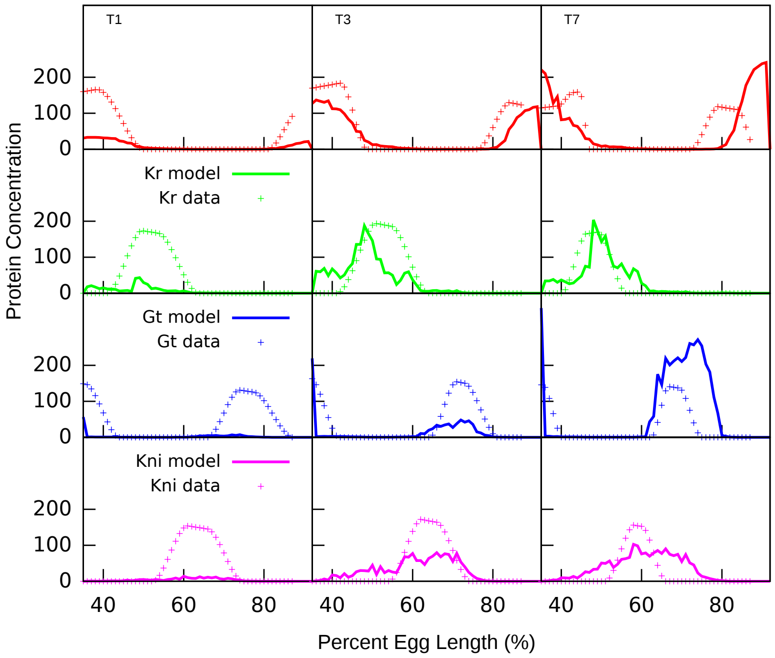

Figure S17: Output for model 4 w/o D as compared to mRNA concentration profiles from the SuperFly. Results are shown for 3 time moments – early (T1), middle (T3) and late (T7) cleavage cycle 14A.

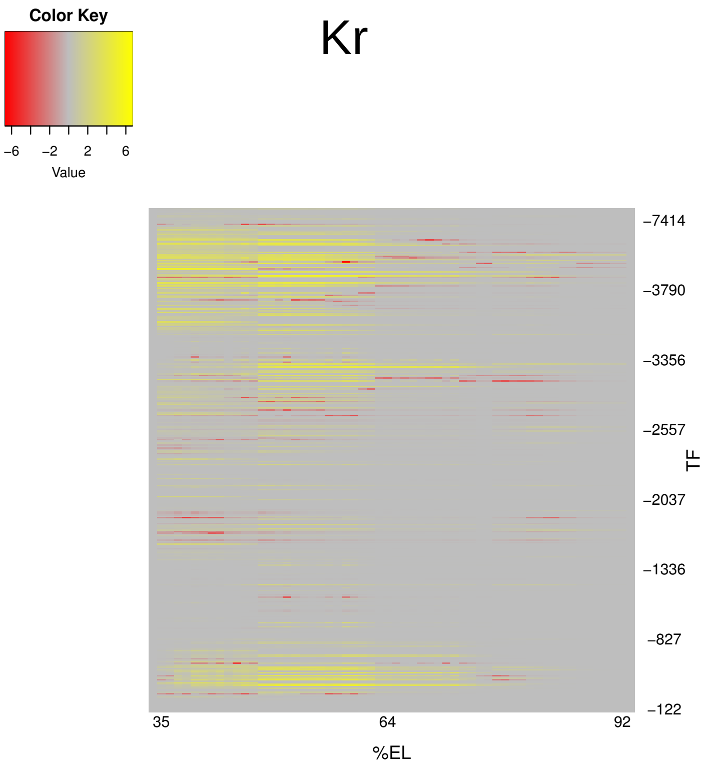

Figure S18: Spatial distribution of impact on gap gene expression patterns of each TFBS in the Kr regulatory region at tempolal class 8 (model 4). The sites are ordered according to their coordinate. Sites from different parts of the regulatory region modulate expression in different spatial locations. Some functionally important sites are arranged in clusters.

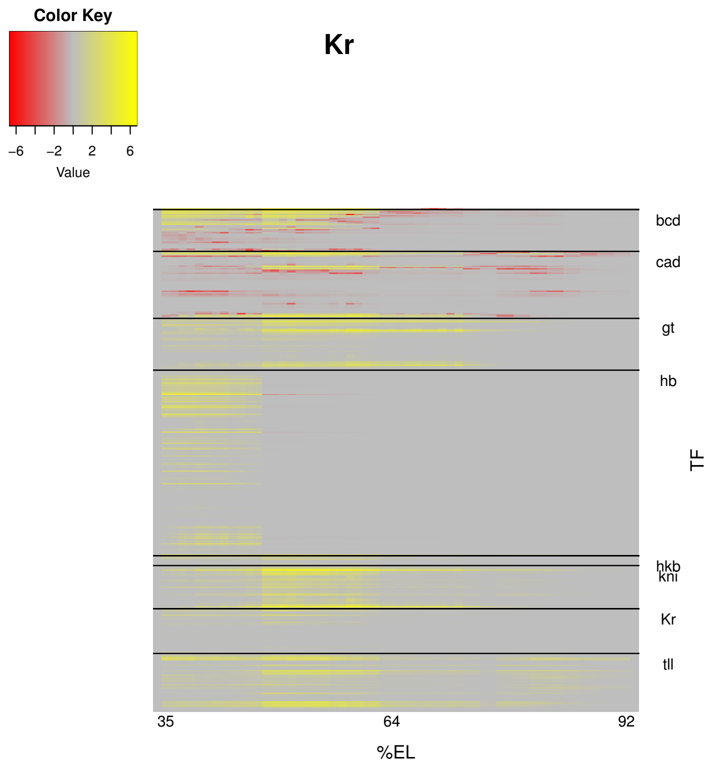

Figure S19: Spatial distribution of impact on gap gene expression patterns of each TFBS in the Kr regulatory region for T8 (model 4). The sites are ordered according to the TF and then by coordinate. Different sites of the same TF may have different spacial effects in the model.

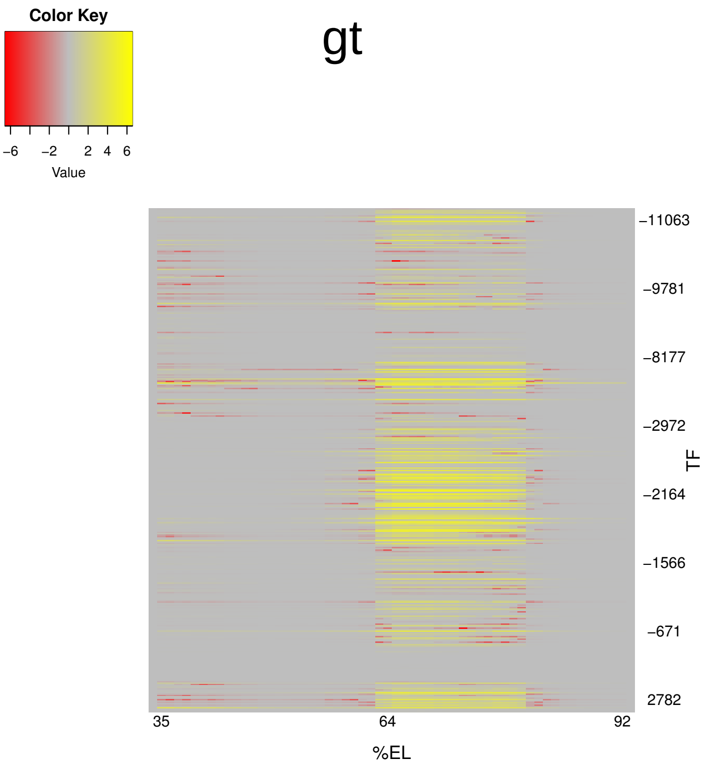

Figure S20: Spatial distribution of impact on gap gene expression patterns of each TFBS in the gt regulatory region at tempolal class 8 (model 4). The sites are ordered according to their coordinate. Sites from different parts of the regulatory region modulate expression in different spatial locations. Some functionally important sites are arranged in clusters.

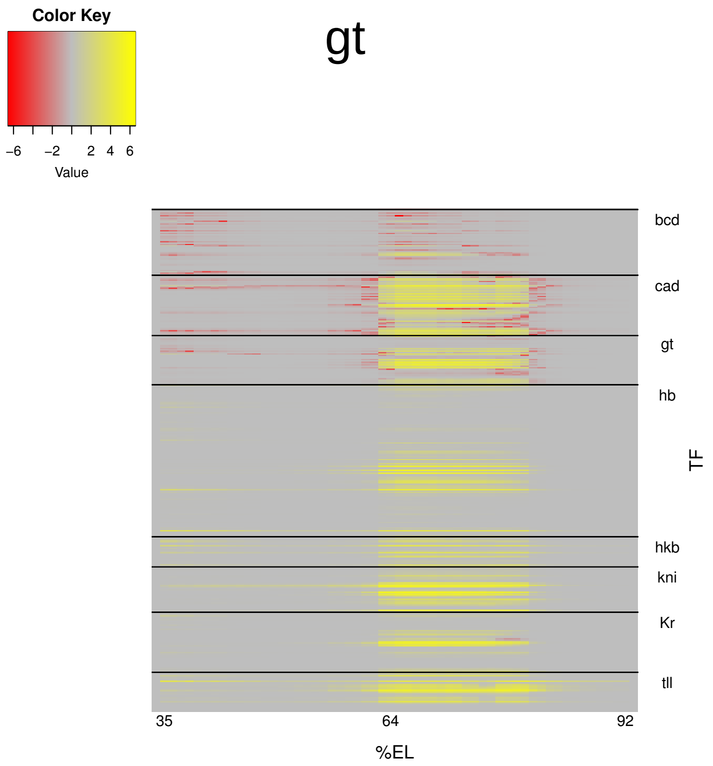

Figure S21: Spatial distribution of impact on gap gene expression patterns of each TFBS in the gt regulatory region for T8 (model 4). The sites are ordered according to the TF and then by coordinate. Different sites of the same TF may have different spacial effects in the model.

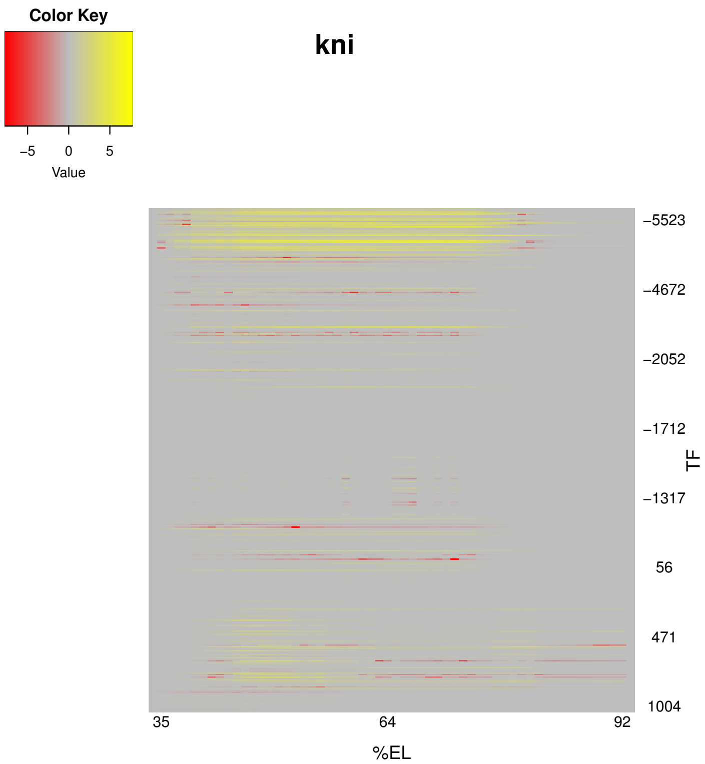

Figure S22: Spatial distribution of impact on gap gene expression patterns of each TFBS in the kni regulatory region at tempolal class 8 (model 4). The sites are ordered according to their coordinate. Sites from different parts of the regulatory region modulate expression in different spatial locations. Some functionally important sites are arranged in clusters.

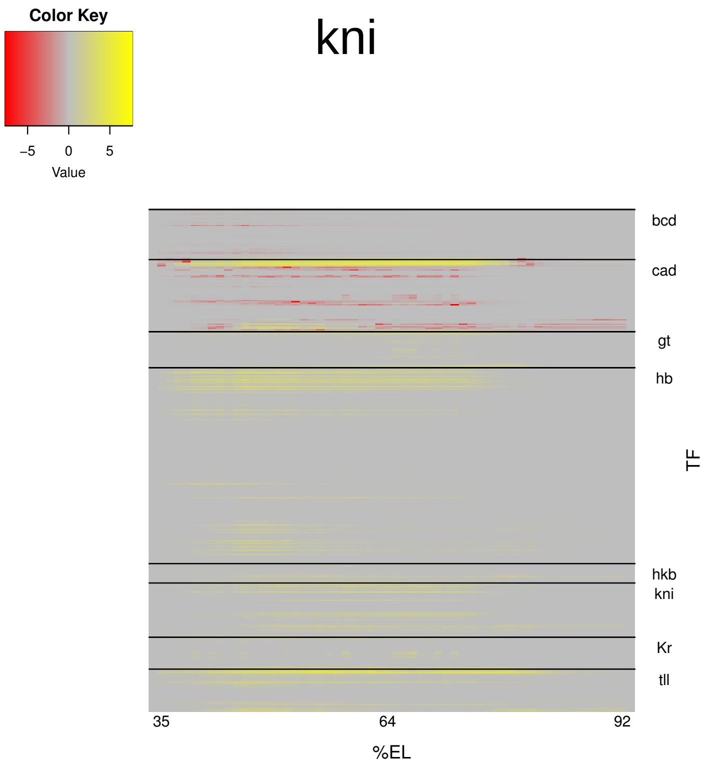

Figure S23: Spatial distribution of impact on gap gene expression patterns of each TFBS in the kni regulatory region for T8 (model 4). The sites are ordered according to the TF and then by coordinate. Different sites of the same TF may have different spacial effects in the model.

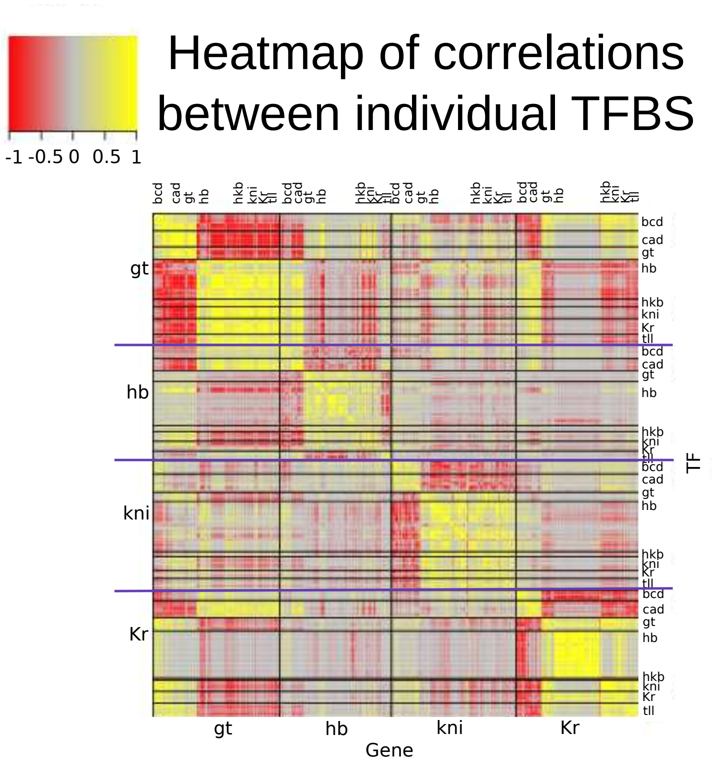

Figure S24: Correlation matrix between spatio-temporal distributions of TFBS impacts (model 4). The diffusion rate parameter was set to zero during calculation. The color in the figure reflects the correlation strength between impact distributions for each pair of TFBSs. The sites are ordered alphabetically – first by target gene (*gt*, *hb*, *kni*, and *Kr*) and then by TF (Bcd, Cad, Gt, Hb, Hkb, Kni, Kr, and Tll) in each group. The clusters of highly correlated sites appear as rectangles of yellow or red color.

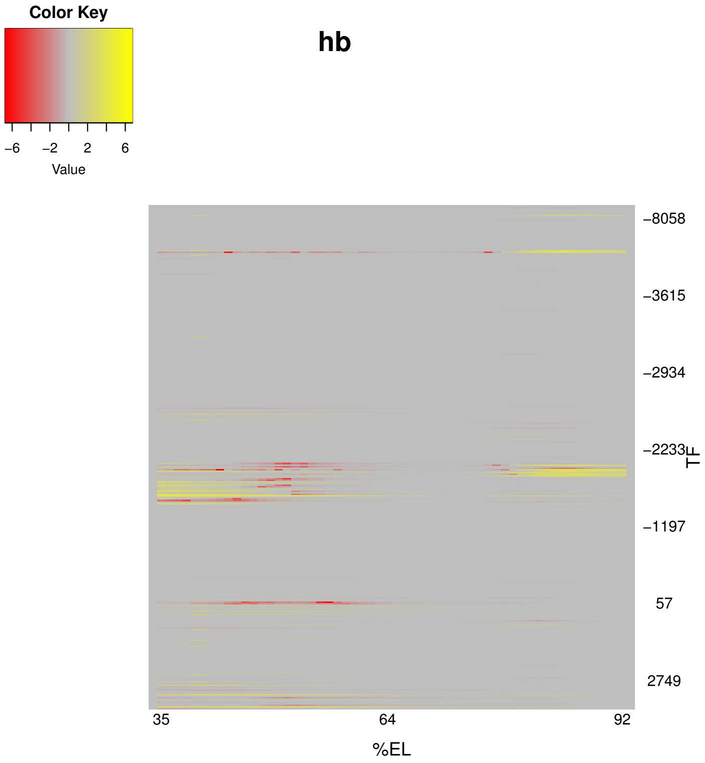

Figure S25: Spatial distribution of impact on gap gene expression patterns of each TFBS in the hb regulatory region at tempolal class 8 (model 3). The sites are ordered according to their coordinate. Sites from different parts of the regulatory region modulate expression in different spatial locations. Some functionally important sites are arranged in clusters.

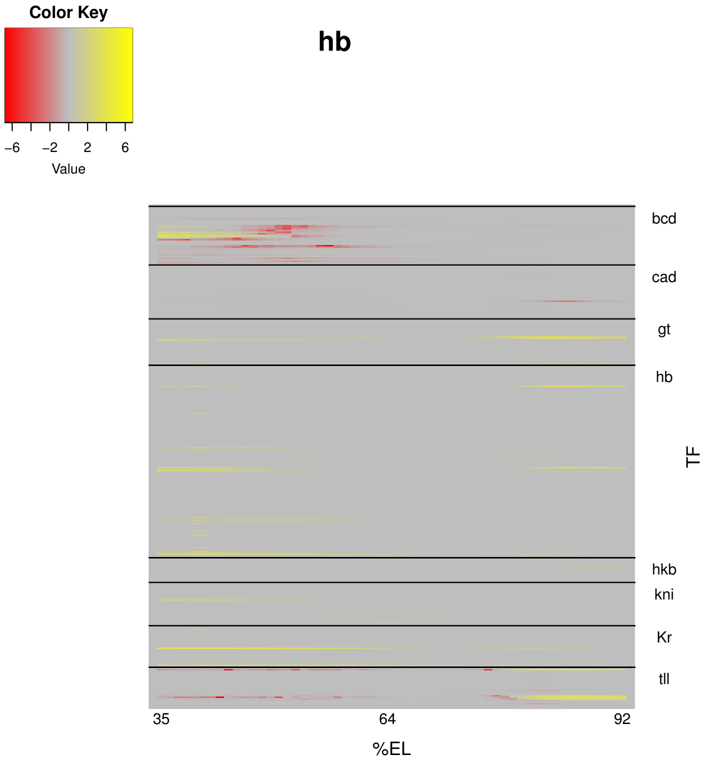

Figure S26: Spatial distribution of impact on gap gene expression patterns of each TFBS in the hb regulatory region for T8 (model 3). The sites are ordered according to the TF and then by coordinate. Different sites of the same TF may have different spacial effects in the model.

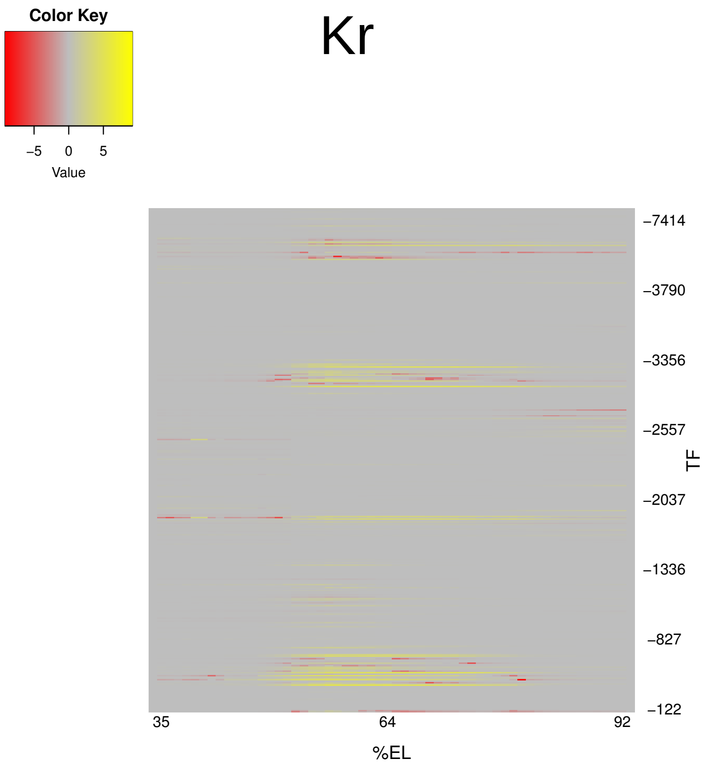

Figure S27: Spatial distribution of impact on gap gene expression patterns of each TFBS in the Kr regulatory region at tempolal class 8 (model 3). The sites are ordered according to their coordinate. Sites from different parts of the regulatory region modulate expression in different spatial locations. Some functionally important sites are arranged in clusters.

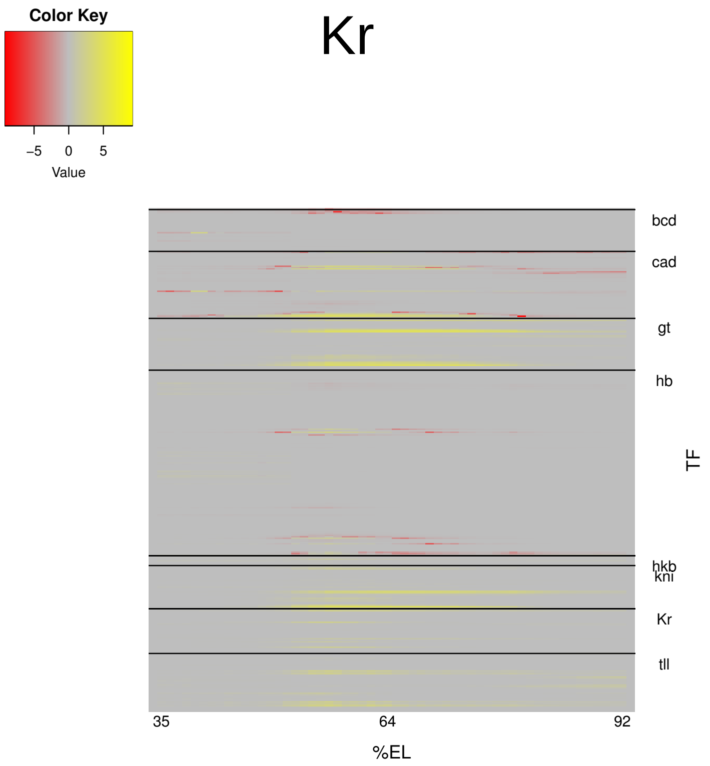

Figure S28: Spatial distribution of impact on gap gene expression patterns of each TFBS in the Kr regulatory region for T8 (model 3). The sites are ordered according to the TF and then by coordinate. Different sites of the same TF may have different spacial effects in the model.

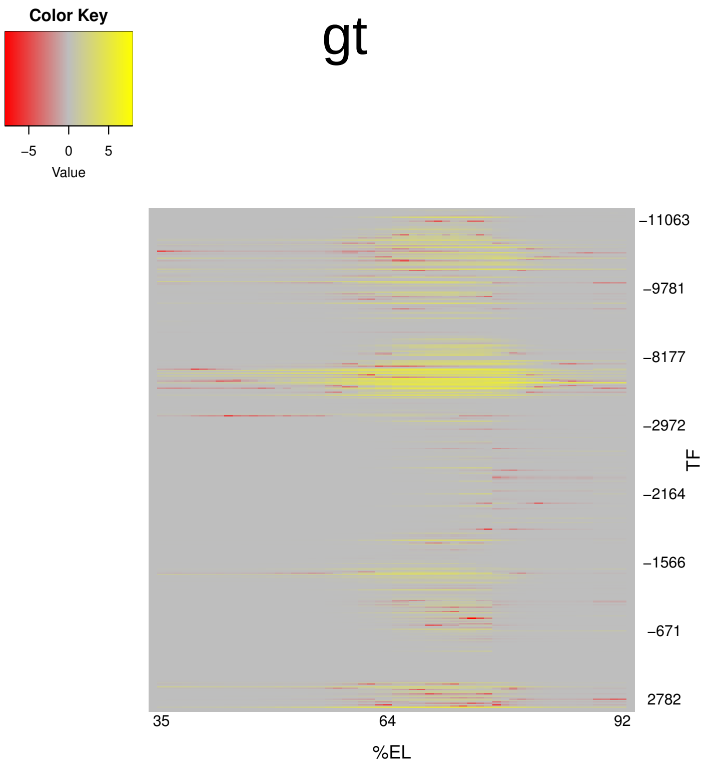

Figure S29: Spatial distribution of impact on gap gene expression patterns of each TFBS in the gt regulatory region at tempolal class 8 (model 3). The sites are ordered according to their coordinate. Sites from different parts of the regulatory region modulate expression in different spatial locations. Some functionally important sites are arranged in clusters.

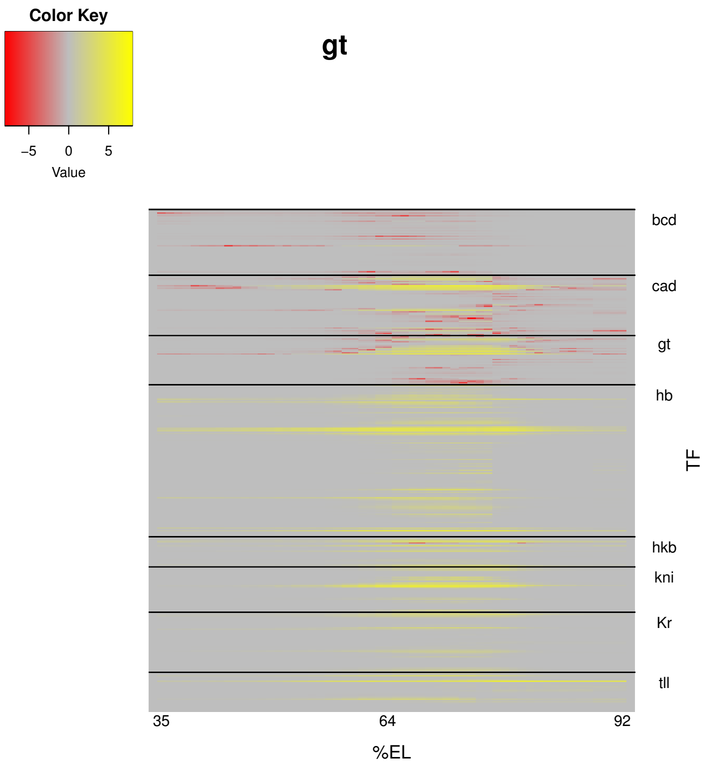

Figure S30: Spatial distribution of impact on gap gene expression patterns of each TFBS in the gt regulatory region for T8 (model 3). The sites are ordered according to the TF and then by coordinate. Different sites of the same TF may have different spacial effects in the model.

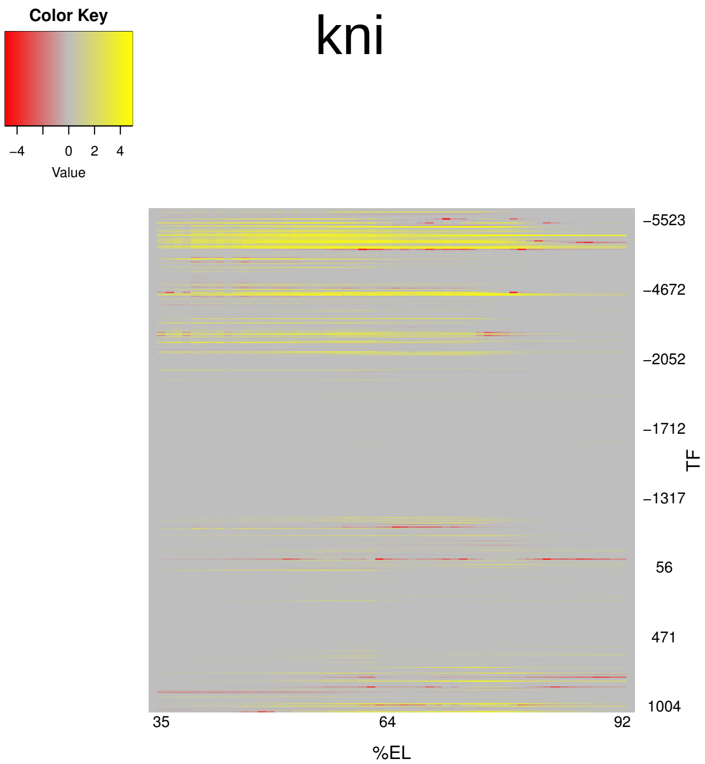

Figure S31: Spatial distribution of impact on gap gene expression patterns of each TFBS in the kni regulatory region at tempolal class 8 (model 3). The sites are ordered according to their coordinate. Sites from different parts of the regulatory region modulate expression in different spatial locations. Some functionally important sites are arranged in clusters.

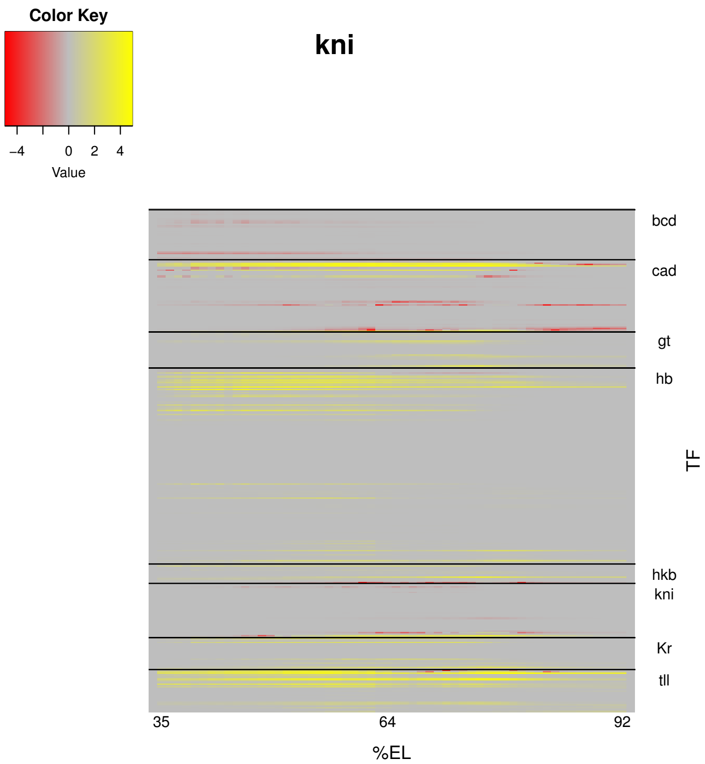

Figure S32: Spatial distribution of impact on gap gene expression patterns of each TFBS in the kni regulatory region for T8 (model 3). The sites are ordered according to the TF and then by coordinate. Different sites of the same TF may have different spacial effects in the model.

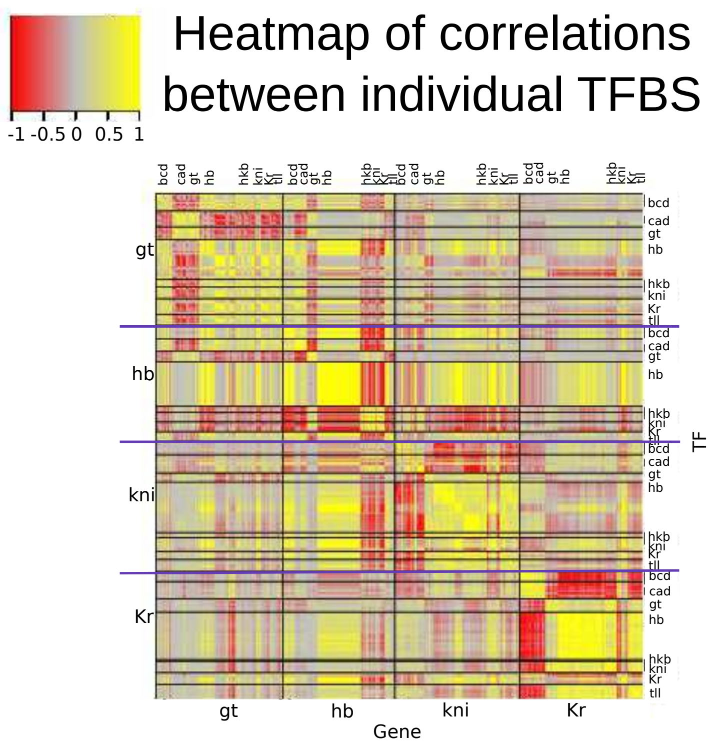

Figure S33: Correlation matrix between spatio-temporal distributions of TFBS impacts (model 2). The diffusion rate parameter was set to zero during calculation. The color in the figure reflects the correlation strength between impact distributions for each pair of TFBSs. The sites are ordered alphabetically – first by target gene (*gt*, *hb*, *kni*, and *Kr*) and then by TF (Bcd, Cad, Gt, Hb, Hkb, Kni, Kr, and Tll) in each group. The clusters of highly correlated sites appear as rectangles of yellow or red color.

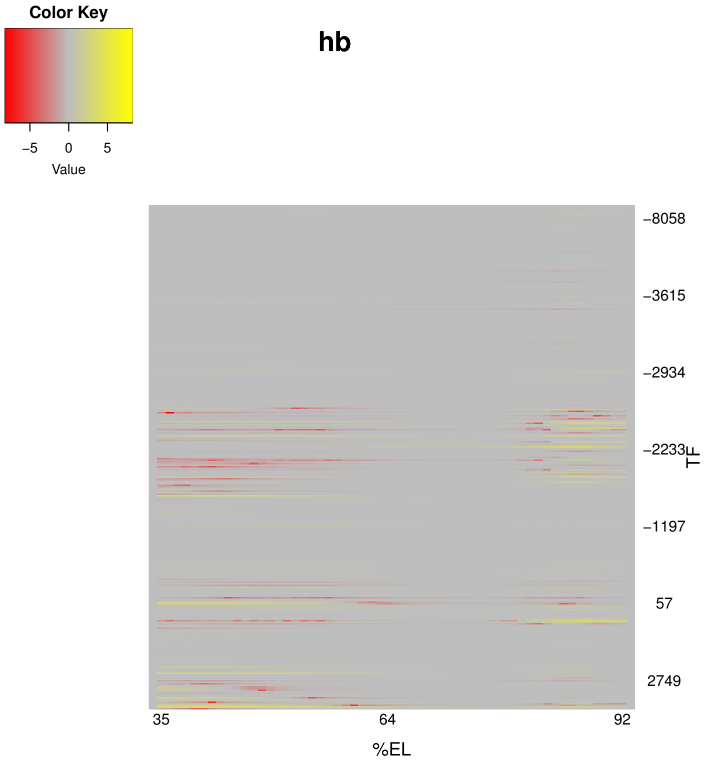

Figure S34: Spatial distribution of impact on gap gene expression patterns of each TFBS in the hb regulatory region at tempolal class 8 (model 2). The sites are ordered according to their coordinate. Sites from different parts of the regulatory region modulate expression in different spatial locations. Some functionally important sites are arranged in clusters.

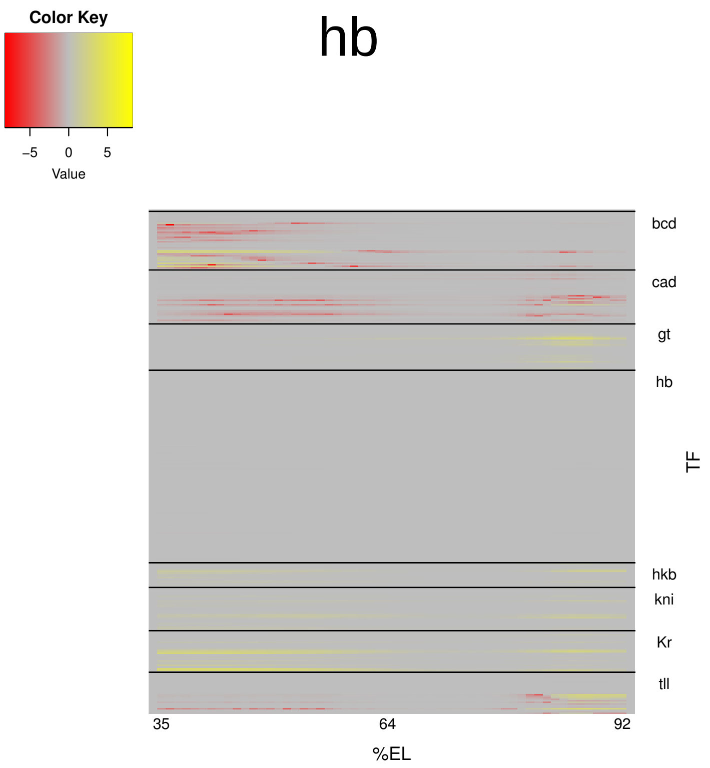

Figure S35: Spatial distribution of impact on gap gene expression patterns of each TFBS in the hb regulatory region for T8 (model 2). The sites are ordered according to the TF and then by coordinate. Different sites of the same TF may have different spacial effects in the model.

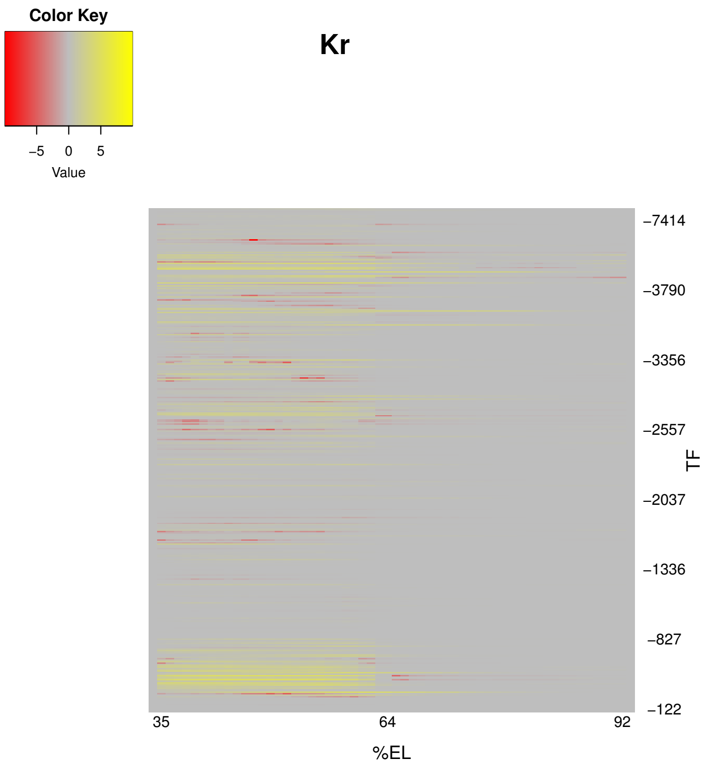

Figure S36: Spatial distribution of impact on gap gene expression patterns of each TFBS in the Kr regulatory region at tempolal class 8 (model 2). The sites are ordered according to their coordinate. Sites from different parts of the regulatory region modulate expression in different spatial locations. Some functionally important sites are arranged in clusters.

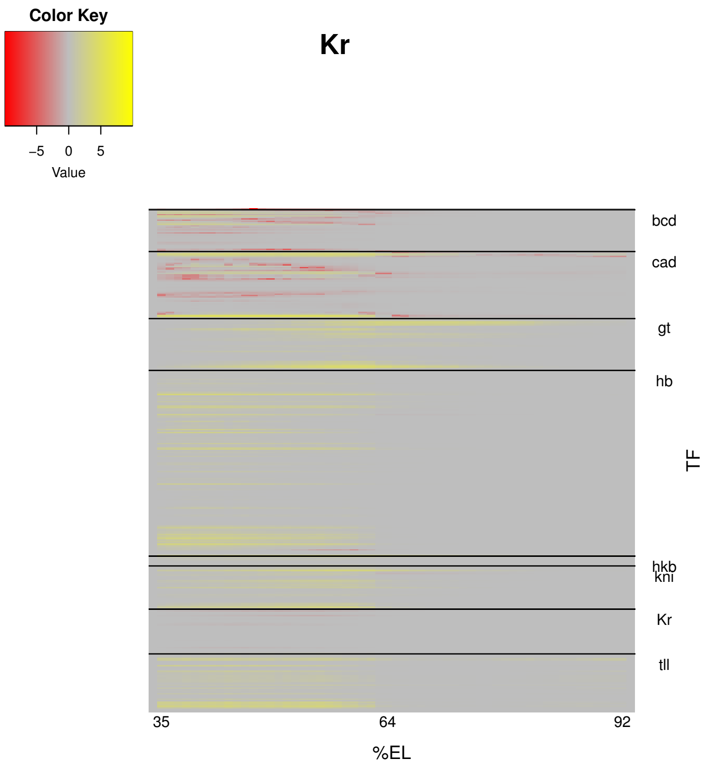

Figure S37: Spatial distribution of impact on gap gene expression patterns of each TFBS in the Kr regulatory region for T8 (model 2). The sites are ordered according to the TF and then by coordinate. Different sites of the same TF may have different spacial effects in the model.

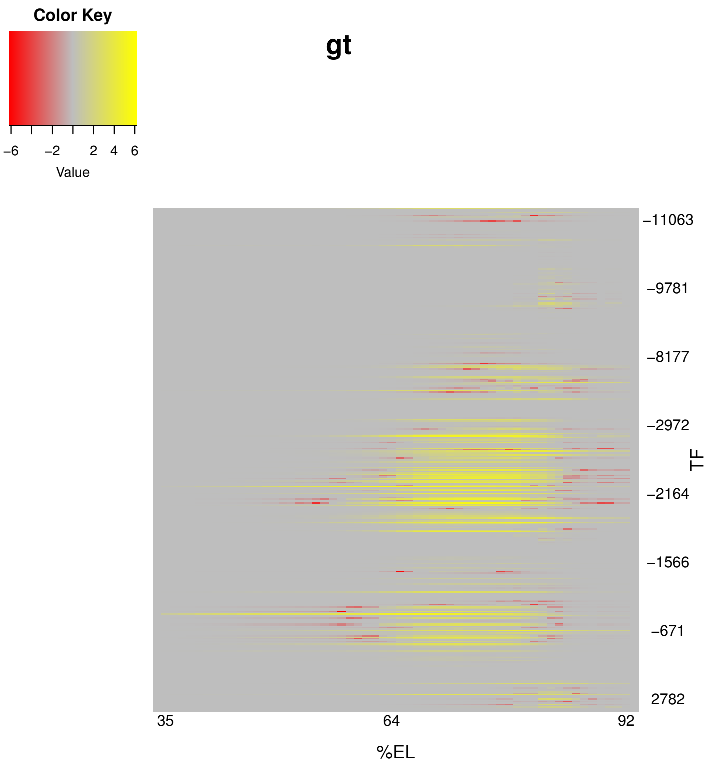

Figure S38: Spatial distribution of impact on gap gene expression patterns of each TFBS in the gt regulatory region at tempolal class 8 (model 2). The sites are ordered according to their coordinate. Sites from different parts of the regulatory region modulate expression in different spatial locations. Some functionally important sites are arranged in clusters.

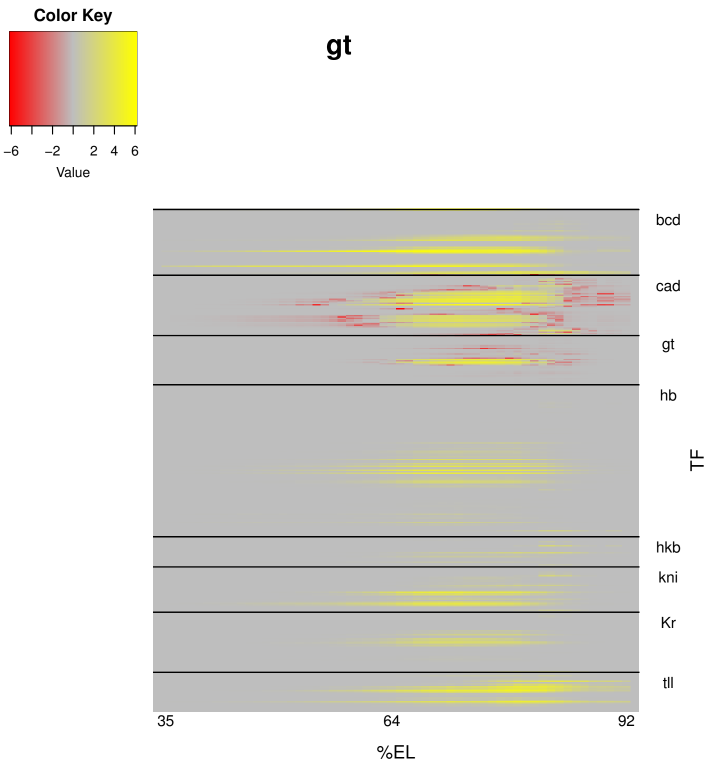

Figure S39: Spatial distribution of impact on gap gene expression patterns of each TFBS in the gt regulatory region for T8 (model 2). The sites are ordered according to the TF and then by coordinate. Different sites of the same TF may have different spacial effects in the model.

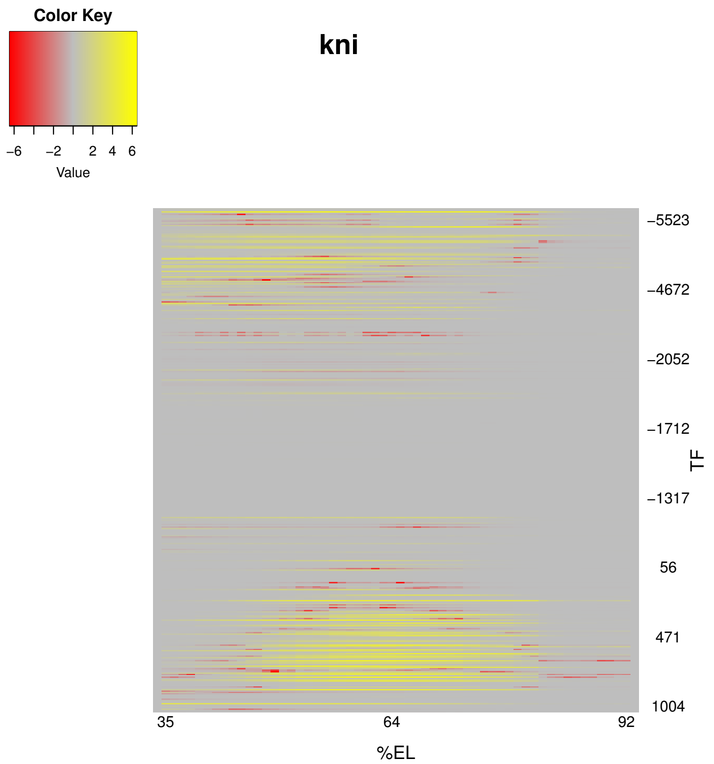

Figure S40: Spatial distribution of impact on gap gene expression patterns of each TFBS in the kni regulatory region at tempolal class 8 (model 2). The sites are ordered according to their coordinate. Sites from different parts of the regulatory region modulate expression in different spatial locations. Some functionally important sites are arranged in clusters.

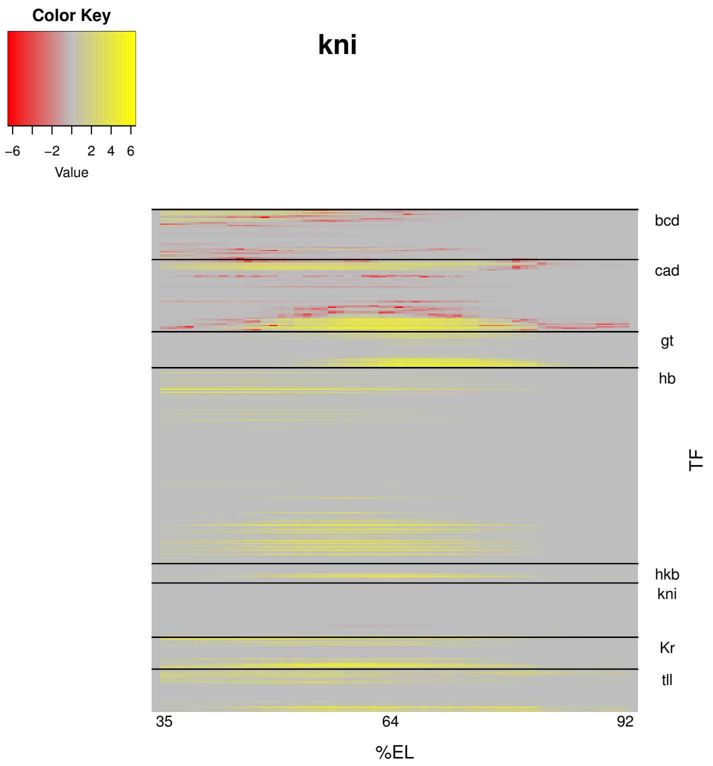

Figure S41: Spatial distribution of impact on gap gene expression patterns of each TFBS in the kni regulatory region for T8 (model 2). The sites are ordered according to the TF and then by coordinate. Different sites of the same TF may have different spacial effects in the model.

References

[1] I.V. Kulakovskiy and V.J. Makeev. Discovery of dna motifs recognized by transcription factors through integration of different experimental sources. *Biophysics*, 54(6):667–674, 2009.
