# Supplementary figures and images for: Analysis of functional importance of binding sites in the Drosophila gap gene network model
Source: BMC Genomics. 2015 Dec 16;16(Suppl 13):S7. doi: 10.1186/1471-2164-16-S13-S7 (PMC4686791; doi:10.1186/1471-2164-16-S13-S7)

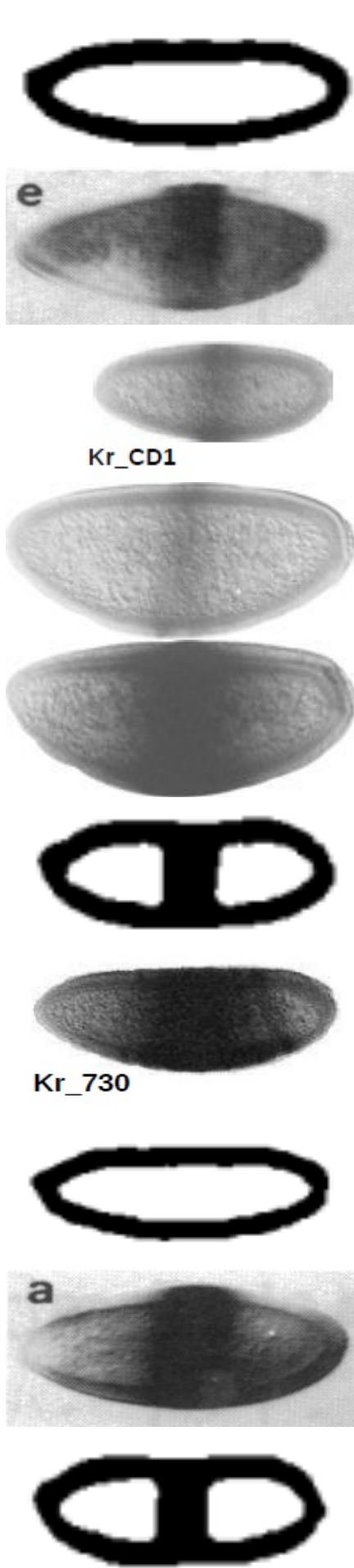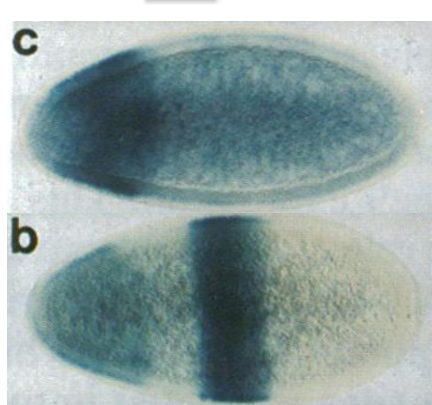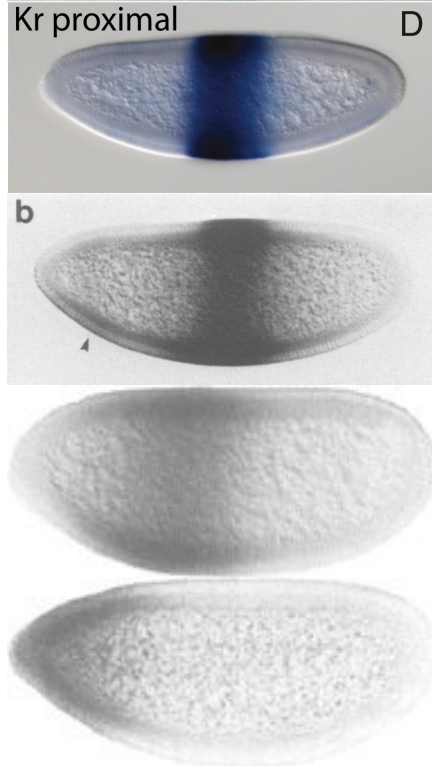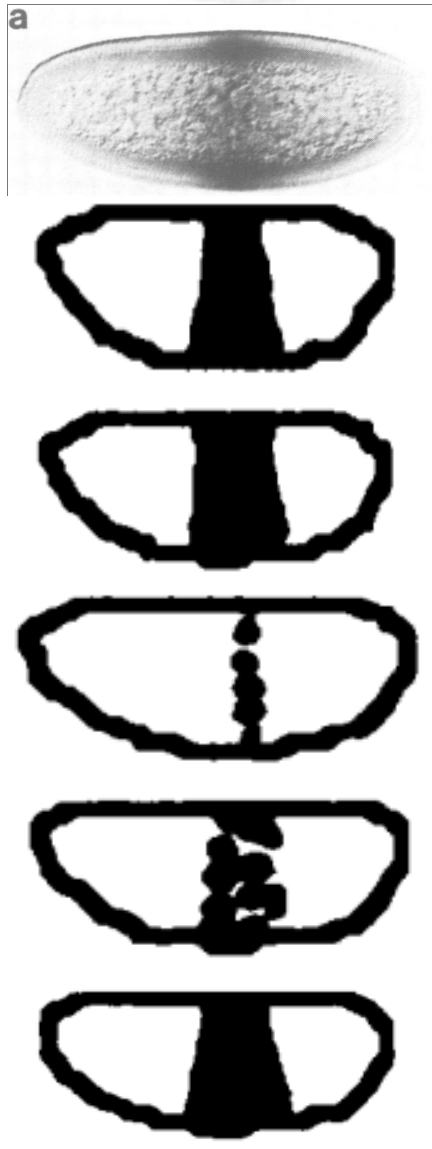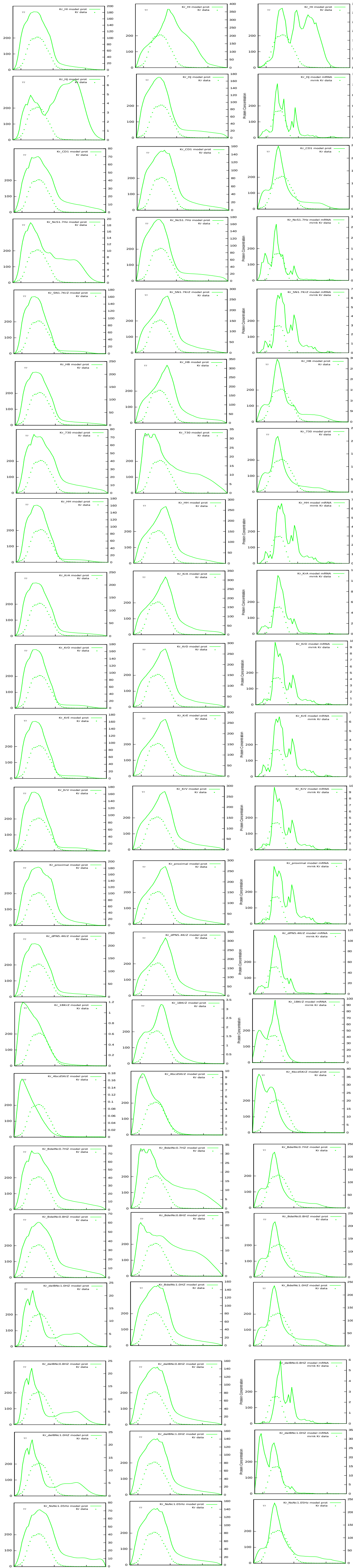

Supplement: Additional file 3 — Supporting Information. Comparison figures. [file 1471-2164-16-S13-S7-S3.PDF]

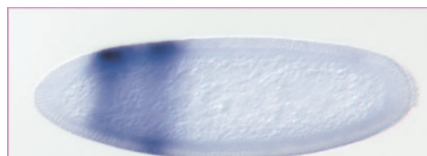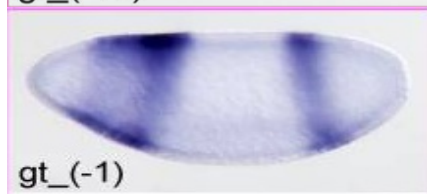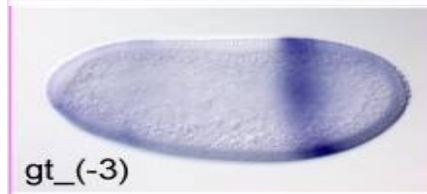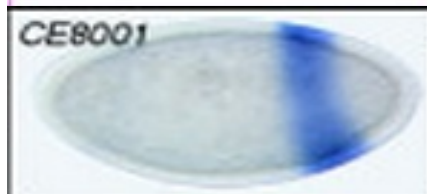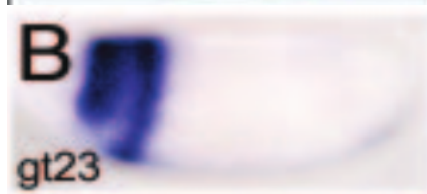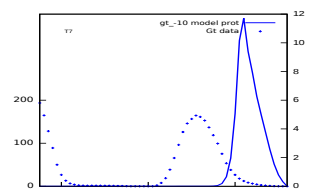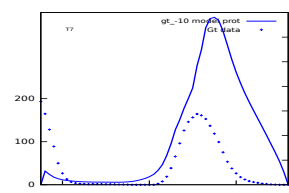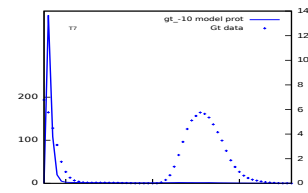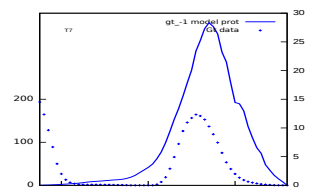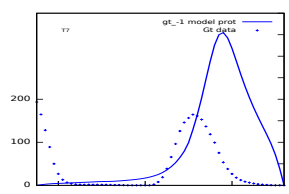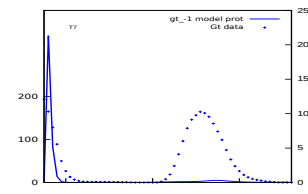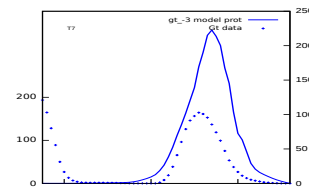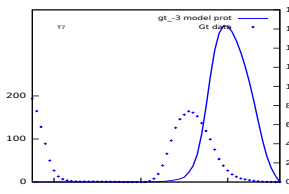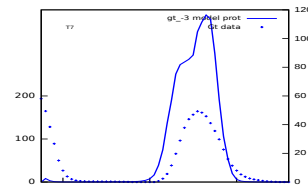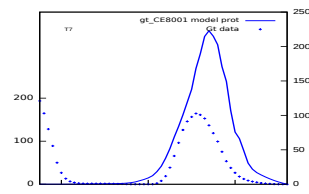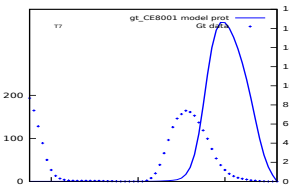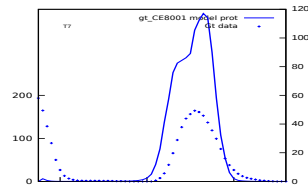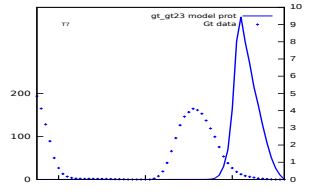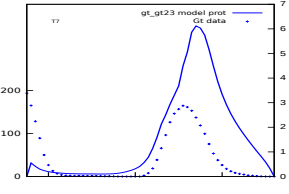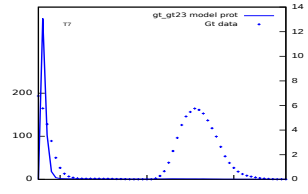

Supplement: Additional file 4 — Supporting Information. Comparison figures. [file 1471-2164-16-S13-S7-S4.PDF]

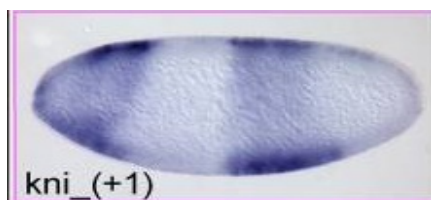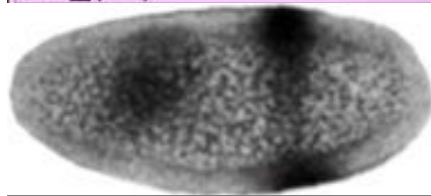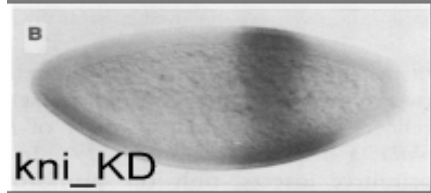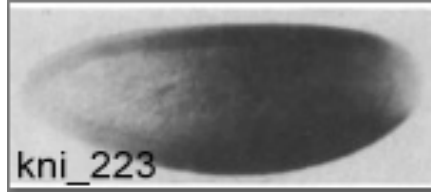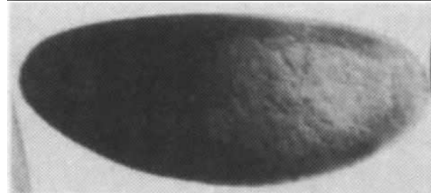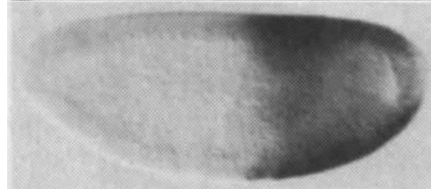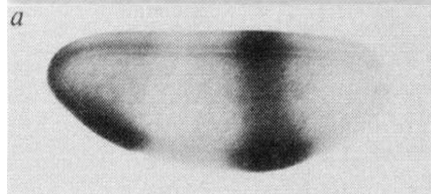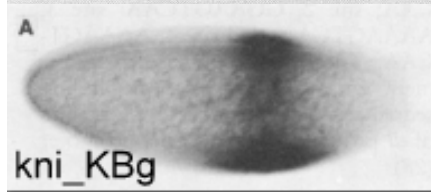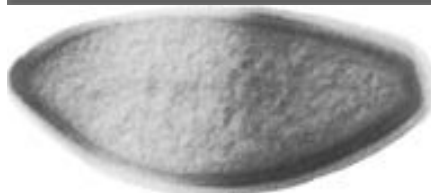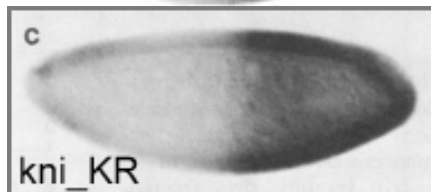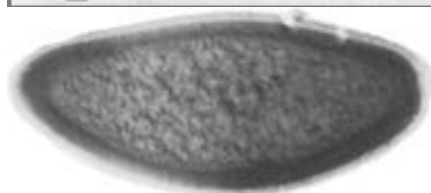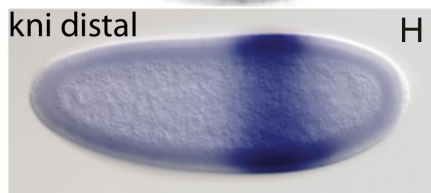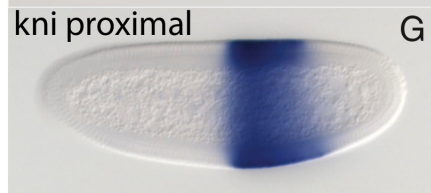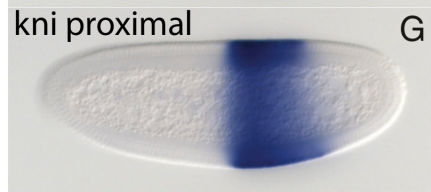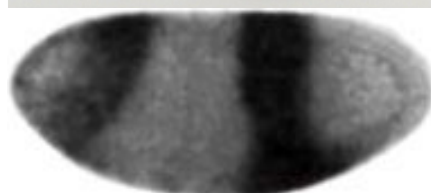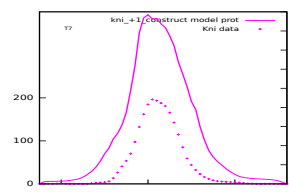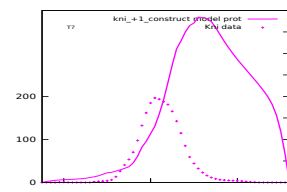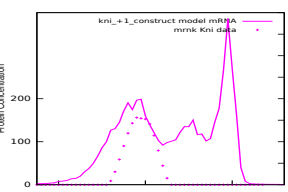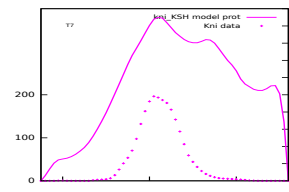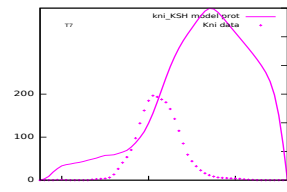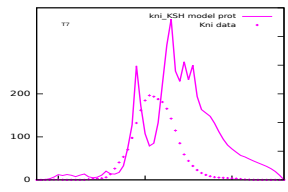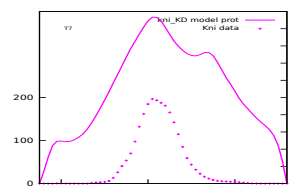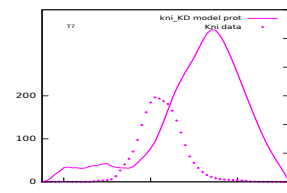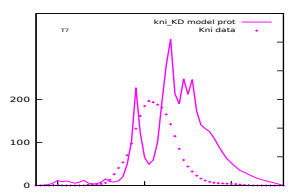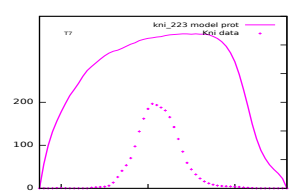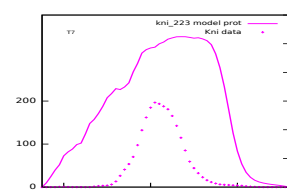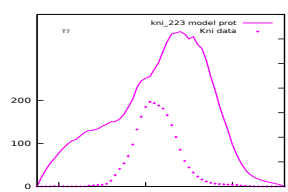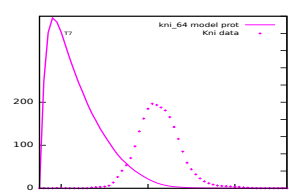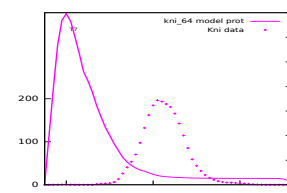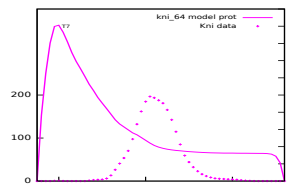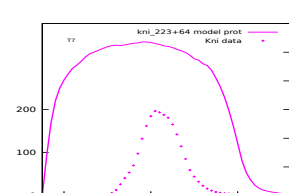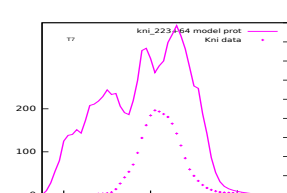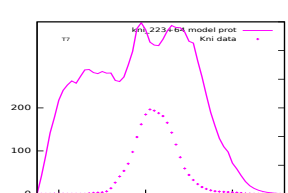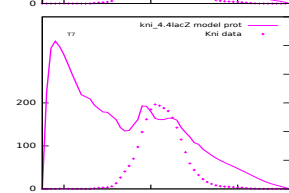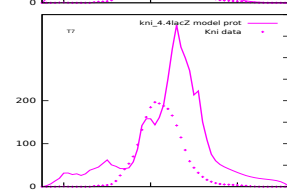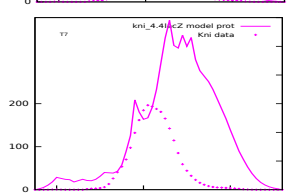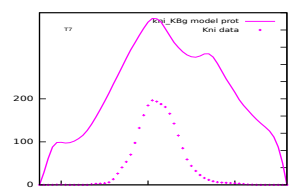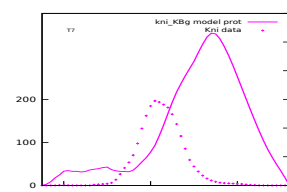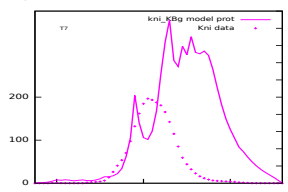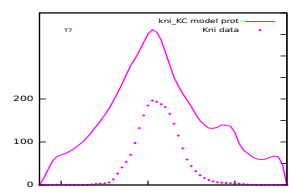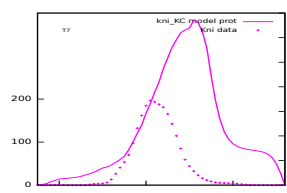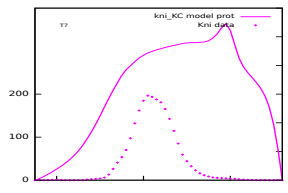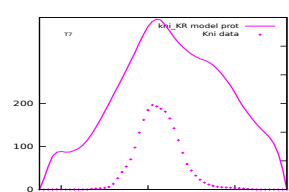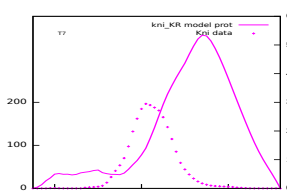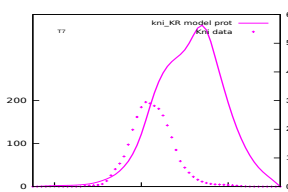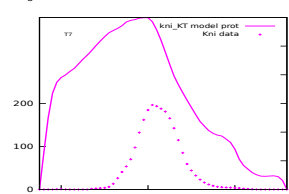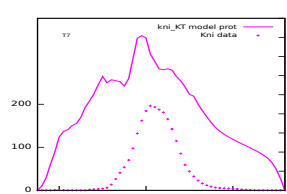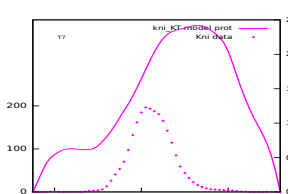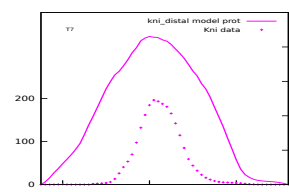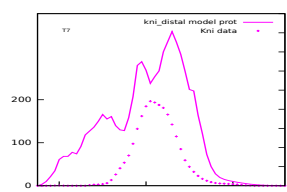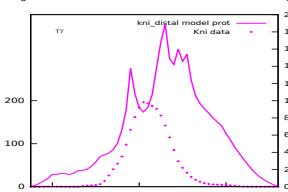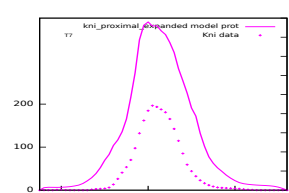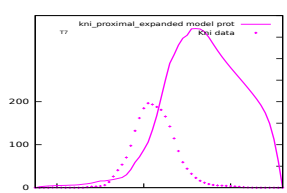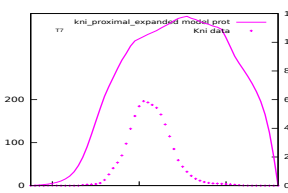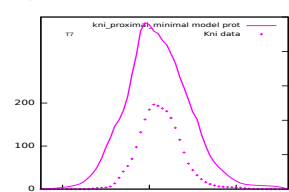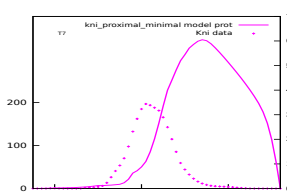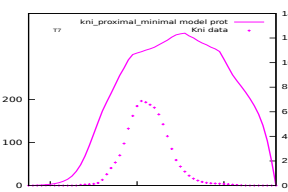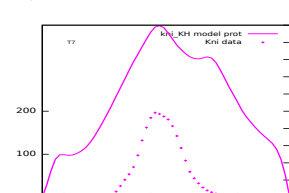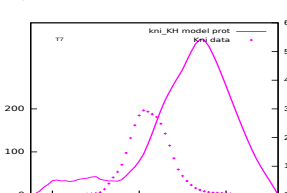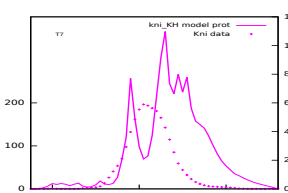

Supplement: Additional file 5 — Supporting Information. Comparison figures. [file 1471-2164-16-S13-S7-S5.PDF]
