# Supplementary material for: Analysis of functional importance of binding sites in the Drosophila gap gene network model
Source: BMC Genomics. 2015 Dec 16;16(Suppl 13):S7. doi: 10.1186/1471-2164-16-S13-S7 (PMC4686791; doi:10.1186/1471-2164-16-S13-S7)
Supplement: Additional file 2 — Supporting Information. Comparison figures. [file 1471-2164-16-S13-S7-S2.PDF]

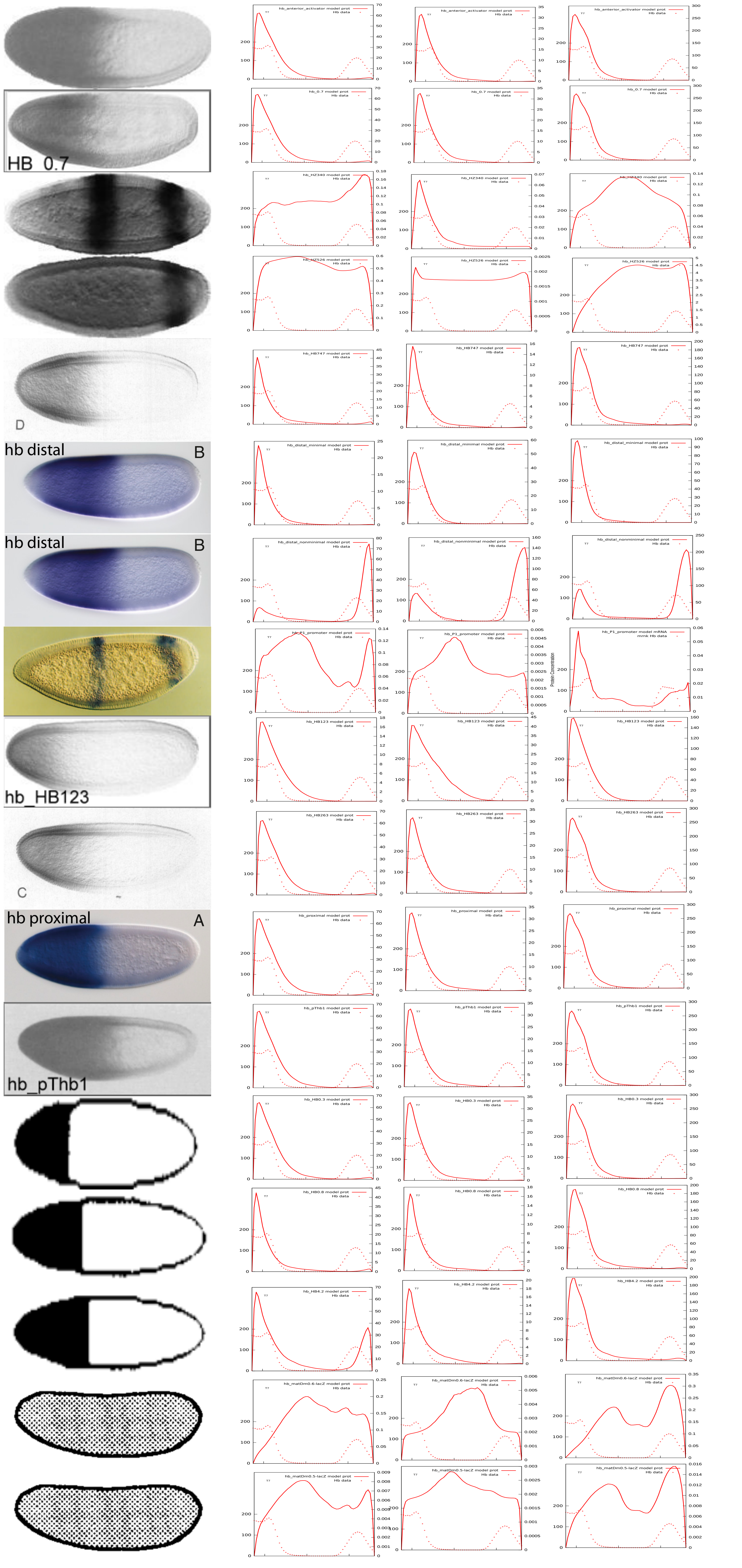
HB_0.7
D
hb distal
B
hb distal
B
hb_HB123
C
hb proximal
A
hb_pThb1
